# The B56γ3-containing protein phosphatase 2A attenuates p70S6K-mediated negative feedback loop to enhance AKT-facilitated epithelial-mesenchymal transition in colorectal cancer

**DOI:** 10.1186/s12964-023-01182-5

**Published:** 2023-07-10

**Authors:** Kai-Ching Hsiao, Siou-Ying Ruan, Shih-Min Chen, Tai-Yu Lai, Ren-Hao Chan, Yan-Ming Zhang, Chien-An Chu, Hung-Chi Cheng, Hung-Wen Tsai, Yi-Fang Tu, Brian K. Law, Ting-Tsung Chang, Nan-Haw Chow, Chi-Wu Chiang

**Affiliations:** 1 Institute of Molecular Medicine, College of Medicine, National Cheng Kung University, Tainan, Taiwan, ROC; 2 Institute of Basic Medical Sciences, College of Medicine, National Cheng Kung University, Tainan, Taiwan, ROC; 3 Department of Surgery, National Cheng Kung University Hospital, College of Medicine, National Cheng Kung University, Tainan, Taiwan, ROC; 4 Department of Pathology, College of Medicine, National Cheng Kung University, Tainan, Taiwan, ROC; 5 Department of Biochemistry and Molecular Biology, College of Medicine, National Cheng Kung University, Tainan, Taiwan, ROC; 6 Department of Pediatrics, National Cheng Kung University Hospital, College of Medicine, National Cheng Kung University, Tainan, Taiwan, ROC; 7 Department of Pharmacology and Therapeutics and the UF-Health Cancer Center, University of Florida, Gainesville, FL 32610 USA; 8 Department of Internal Medicine, National Cheng Kung University Hospital, College of Medicine, National Cheng Kung University, Tainan, Taiwan, ROC

**Keywords:** AKT, B56γ3, Colorectal cancer, EMT, PP2A, p70S6K

## Abstract

**Background:**

Protein phosphatase 2A (PP2A) is one of the major protein phosphatases in eukaryotic cells and is essential for cellular homeostasis. PP2A is a heterotrimer comprising the dimeric AC core enzyme and a highly variable regulatory B subunit. Distinct B subunits help the core enzyme gain full activity toward specific substrates and contribute to diverse cellular roles of PP2A. PP2A has been thought to play a tumor suppressor and the B56γ3 regulatory subunit was shown to play a key tumor suppressor regulatory subunit of PP2A. Nevertheless, we uncovered a molecular mechanism of how B56γ3 may act as an oncogene in colorectal cancer (CRC).

**Methods:**

Polyclonal pools of CRC cells with stable B56γ3 overexpression or knockdown were generated by retroviral or lentiviral infection and subsequent drug selection. Co-immunoprecipitation(co-IP) and in vitro pull-down analysis were applied to analyze the protein–protein interaction. Transwell migration and invasion assays were applied to investigate the role of B56γ3 in affecting motility and invasive capability of CRC cells. The sensitivity of CRC cells to 5-fluorouracil (5-FU) was analyzed using the PrestoBlue reagent assay for cell viability. Immunohistochemistry (IHC) was applied to investigate the expression levels of phospho-AKT and B56γ3 in paired tumor and normal tissue specimens of CRC. DataSets of TCGA and GEO were analyzed to investigate the correlation of B56γ3 expression with overall survival rates of CRC patients.

**Results:**

We showed that B56γ3 promoted epithelial-mesenchymal transition (EMT) and reduced the sensitivity of CRC cells to 5-FU through upregulating AKT activity. Mechanistically, B56γ3 upregulates AKT activity by targeting PP2A to attenuate the p70S6K-mediated negative feedback loop regulation on PI3K/AKT activation. B56γ3 was highly expressed and positively correlated with the level of phospho-AKT in tumor tissues of CRC. Moreover, high B56γ3 expression is associated with poor prognosis of a subset of patients with CRC.

**Conclusions:**

Our finding reveals that the B56γ3 regulatory subunit-containing PP2A plays an oncogenic role in CRC cells by sustaining AKT activation through suppressing p70S6K activity and suggests that the interaction between B56γ3 and p70S6K may serve as a therapeutic target for CRC.

Video Abstract

**Supplementary Information:**

The online version contains supplementary material available at 10.1186/s12964-023-01182-5.

## Background

PP2A is an abundant serine/threonine protein phosphatase in mammalian cells and can contribute near 1% of total cellular proteins in some cell types. PP2A plays a role in regulating essential cellular functions, ranging from metabolism, growth, to apoptosis [1, 2]. The PP2A holoenzyme consists of a dimeric core enzyme, which includes a catalytic subunit (C) and a structural subunit (A), and a variable regulatory subunit (B). The assembly of variable B regulatory subunits with the PP2A core enzyme not only controls the substrate specificity and subcellular localization of PP2A holoenzymes, but also gives rise to the structural and functional complexity of PP2A [3]. Four distinct B subunit families have been identified, including B (B55 or PR55), B’ (B56 or PR61), B’’ (PR72) and B’’’ (PR93/PR110), and the individual B subunits are differentially expressed in tissues, cells, and are located in distinct subcellular compartments [1]. PP2A has been thought to play a tumor suppressor role in cells and diverse B regulatory subunits have been shown to confer tumor-suppressive activity to PP2A by targeting the PP2A holoenzyme to regulate substrates participating in the process of tumor progression [4, 5]. The PI3K/AKT/mTOR signaling pathway transduces signaling downstream of growth factors and nutrients to regulate metabolism, growth, and proliferation [6]. Dysregulation of the PI3K/AKT/mTOR signaling axis caused by hyperactivation and mutation of the molecules involved in this signaling pathway is often found in a variety of tumors [7, 8]. The 70-kDa ribosomal protein S6 kinase p70S6K functions as a crucial integrator of growth factors and nutrient signals downstream of mTOR to regulate translation and protein synthesis [6]. p70S6K is associated with tumorigenesis and plays a key role in tumor initiation and progression in a variety of cancer types [9, 10]. Importantly, growth factor-activated p70S6K is also responsible for mediating a negative feedback loop to attenuate growth factor-stimulated RTK/PI3K/AKT signaling and uncontrolled cell growth [11, 12].

PP2A interacts with and counterbalances the PI3K/AKT/mTOR signaling pathway to control cell growth and survival [13, 14]. In addition, PP2A has long been known to regulate the activity of p70S6K and to catalyze the dephosphorylation of phospho-Thr229 and phospho-Thr389, which are key phosphorylation sites for p70S6K activity [15–17]. In addition, the Drosophila B' regulatory subunit of PP2A specifically targets the PP2A holoenzyme to dephosphorylate S6K and regulate insulin signaling [18]. Moreover, knockdown of PPP2R5C, which encodes several variants of the B56γ subunit, was shown to increase phosphorylation of p70S6K at Thr389 in human cells [18]. However, it remains unclear whether a specific B56γ subunit is directly responsible for targeting PP2A holoenzyme to regulate p70S6K.

Among the B56γ variants, B56γ3 was shown to play a critical role in inhibiting transformation of HEK cells expressing LT, hTERT, and Ras-V12 and in attenuating tumorigenicity of human lung cancer cell lines [19]. B56γ3 also targets the PP2A holoenzyme to regulate the level and activity of key cell cycle regulators, such as p53 and p27Kip, to control cell proliferation [20–22]. Here, we delineated the role and molecular mechanism of the B56γ3-containing PP2A holoenzyme (PP2A-B56γ3) in regulating the AKT/mTOR/p70S6K signaling pathway. Intriguingly, we found that B56γ3 positively regulated AKT phosphorylation while negatively regulated p70S6K phosphorylation in several cell lines. We further revealed the underlying mechanism by which B56γ3 upregulated AKT phosphorylation by attenuating p70S6K-mediated negative feedback activity downstream of growth factor stimulation. In contrast to its well-known tumor suppressor activity, we found that B56γ3 promoted EMT and reduced the drug sensitivity in colorectal cancer cells by enhancing AKT activity. We further demonstrated that the expression of B56γ3 was positively associated with the expression of phospho-AKT in a subset of human colorectal cancer (CRC) tissues and that high B56γ3 expression was associated with poor prognosis in CRC.

## Methods

### DNA constructs

All expression vectors for 4HAB56γ3 were constructed as reported previously [21]. pGEX4T-1-FLAG-p70S6K1 was prepared by excising cDNA encoding the FLAG-p70S6K from pcDNA3.1-FLAG-p70S6K1, which was subsequently cloned into the bacterial expression vector pGEX4T-1 (GE Healthcare). pLKO.1 vector harboring cDNA encoding the B56γ3-specific shRNA (TRCN0000002495) and shLuc (TRCN00000072244) were obtained from the National RNAi Core Facility (Institute of Molecular Biology/Genomic Research Center, Academia Sinica, Taiwan). For PPP2R5C (B56γ) knockout, vectors of gRNAs targeting PPP2R5C, pRP[CRISPR]-PPP2R5C[gRNA#2] and pRP[CRISPR]-PPP2R5C[gRNA#3], were obtained from Vectorbuilder (Taiwan), and the gRNA#2 sequence was 5'-CTGTGATCACATTCCGATTA 3' and the gRNA#3 sequence was 5'-TACGGGAGCGGAATTTGACC-3’.

### Cell culture and cell lines

All cell lines were obtained from ATCC. NIH3T3 and HEK293 were cultured in Dulbecco’s modified Eagle medium (DMEM) (Invitrogen). SW480, DLD-1, and HCT116 cells were cultured in RPMI-1640 medium (Invitrogen) or DMEM/F12 medium (GeneDireX). HeLa and Hep3B cells were cultured in minimal essential medium (MEM)(Invitrogen). All cell lines, except NIH3T3 cultured in DMEM supplemented with bovine serum (BS), were cultured in medium supplemented with 10% fetal bovine serum (FBS, Gibco), L-glutamine (2 mM) (Invitrogen), and penicillin and streptomycin (100 units/ml) (Invitrogen). All cell lines were cultured in 5% CO_2_ incubator at 37℃. For growth factor stimulation, cells were serum starved (0–0.5% serum) for 24 h prior to stimulation with insulin (Sigma-Aldrich) at 100 nM (NIH3T3 and HeLa cells), with EGF (Sigma-Aldrich) at 50 ng/ml or 20 ng/ml (HeLa and HCT116 cells, respectively), or with IGF-1 (Millipore) at 50 ng/ml (SW480 cells). Polyclonal pools of NIH3T3, HeLa, Hep3B, HCT116, SW480 cells stably overexpressing pMSCV4HA-B56γ3 or empty pMSCV vector were created using procedures of retroviral infection and subsequent drug selection as described previously [23]. HeLa, Hep3B, HCT116, SW480 cells stably overexpressing specific shRNA against B56γ3(shB56γ3) (TRCN0000002495) or shLuc (TRCN00000072244) were created by lentiviral infection and subsequent drug selection as described before with some modifications [23]. Briefly, lentiviruses harboring shB56γ3 or shLuc were obtained by collecting of supernatant of HEK293T cells transfected with pLKO.1 vector containing cDNA of shB56γ3 or shLuc, pVSV-G and delta-8.91 vector by calcium phosphate precipitation method.

### Cell viability analysis

Cells were incubated with DMSO or various doses of 5-FU for 72 h and, subsequently, were subject to PrestoBlue™ viability assay (Thermo Fisher Scientific) according to recommend standard procedures. The relative viability was quantitated by the ratio of absorbance readings at OD570 to that at OD600.

### Western blotting and co-immunoprecipitation

Cell lysates for Western blotting were prepared in radioimmunoprecipitation assay (RIPA) buffer and cell lysates for immunoprecipitation (IP) were prepared in isotonic IP buffer as described before [24]. For better transfection efficiency, in addition to CRC cells, we also applied HeLa and HEK293 cells in the co-immunoprecipitation analysis. The procedures of Western blotting and IP were performed according to protocols described before [24]. Details regarding the antibodies used in this study are listed in Supplementary Table 1. Western blots were developed using enhanced chemiluminescence. Results were quantified by densitometry using Alpha Innotech AlphaImager.

### In vitro pull down assay

Recombinant His-B56γ3, GST and GST-Flag-p70S6K WT proteins were prepared as previously described [23]. Two μg of purified recombinant His-B56γ3 proteins were incubated with 2 μg recombinant GST-Flag-p70S6K WT or GST proteins for 4 h at 4 °C. GST or GST-Flag-p70S6K WT were pulled down according to the protocols described before [23].

### In vitro phospho-p70S6K dephosphorylation analysis

The analysis was performed according to the protocol previously described with some modifications [21]. Phosphorylated Flag-p70S6K WT protein was immunoprecipitated using anti-FLAG-agarose from serum-stimulated NIH3T3 cells transfected with vector of Flag-p70S6K WT using transfection reagent Lipofectamine 2000 (Invitrogen). The PP2A-B56γ3 holoenzymes were prepared as described before [21]. Aliquots of phospho-Flag-p70S6K WT were then mixed with various amounts of 4HAB56γ3 immunocomplexes in the phosphatase assay buffer with or without 100 nM OA (Alexis) and incubated at 30 °C for 30 min. The dephosphorylation reactions were terminated by adding 4X SDS sample buffer and boiling for 5 min.

### Knockout of PPP2R5C by the CRISPR/Cas9 approach

Single clones of HCT116 cells with knockout of PPP2R5C were established by co-transfection with pRP[CRISPR]-PPP2R5C[gRNA#2] and pRP[CRISPR]-PPP2R5C[gRNA#3], and selection with puromycin (5 μg/ml) for 3–4 weeks after transfection.

### Transwell migration assay

Cells in the basal medium without adding serum were seeded onto the upper chamber of Transwell Permeable Supports (8.0 μm in membrane pore size, Corning) with lower chamber filled with medium containing 10% FBS. Twenty-four hours after seeding, inserts of Transwell Permeable Supports were then fixed and stained with 0.1% crystal violet and cells that had not migrated were removed using a cotton swab. Migrated cells stained with crystal violet were then photographed using a Nikon ECLIPSE TS100 microscope equipped with a digital camera at 100X magnification. Cells were then counted manually or by Image J.

### Transwell invasion assay

Cells in the basal medium without adding serum were seeded onto the upper chamber of the BioCoat Matrigel Invasion Chamber (8.0 μm in membrane pore size, Corning), which contained a PET membrane with a thin layer of Matrigel basement membrane matrix, with lower chamber filled with medium containing 10% FBS. Twenty-four hours after seeding, inserts of Matrigel Invasion Chamber were then fixed, stained with 0.1% crystal violet and processed as described earlier.

### Immunohistochemistry

Paraffin-embedded paired tumor and normal tissue specimens were obtained from the Human Biobank, Research Center of Clinical Medicine, National Cheng Kung University Hospital. The sections were incubated with anti-B56γ3 antibody (Santa Cruz) or anti-phospho-AKT (Ser473) (Cell Signaling Technology), and avidin–biotin-HRP complexes (Thermo Fisher) in conjunction with biotinylated anti-mouse or anti-rabbit secondary antibodies. DAB + substrate (Thermo Fisher) was used to develop the signals. The tissues were counterstained with hematoxylin. Quantification of immunohistochemical staining was performed using an immunoreactive score (IRS) system [25]. IRS scores = (scores of staining intensity) × (percentage of positive cells). This study was approved by the institutional review board of the National Cheng Kung University Hospital.

### Kaplan–Meier survival curves

Analysis of the overall survival rate of CRC patients stratified by levels of PPP2R5C expression was carried out using TCGA and GEO colon adenocarcinoma (COAD) datasets through PROGgeneV2 database [26]. The grouping of low and high expression level of PPP2R5C is divided by median expression level.

### Statistical analysis

Statistical analyses were performed using two-tailed unpaired Student’s t-test. The contingency of the grouped IHC data was examined using the chi-square test. All data were analyzed using GraphPad Prism 5. In all statistical analyses, a *p*-value of < 0.05 was considered significant.

## Results

### B56γ3 upregulates AKT phosphorylation

**Fig. 1:**
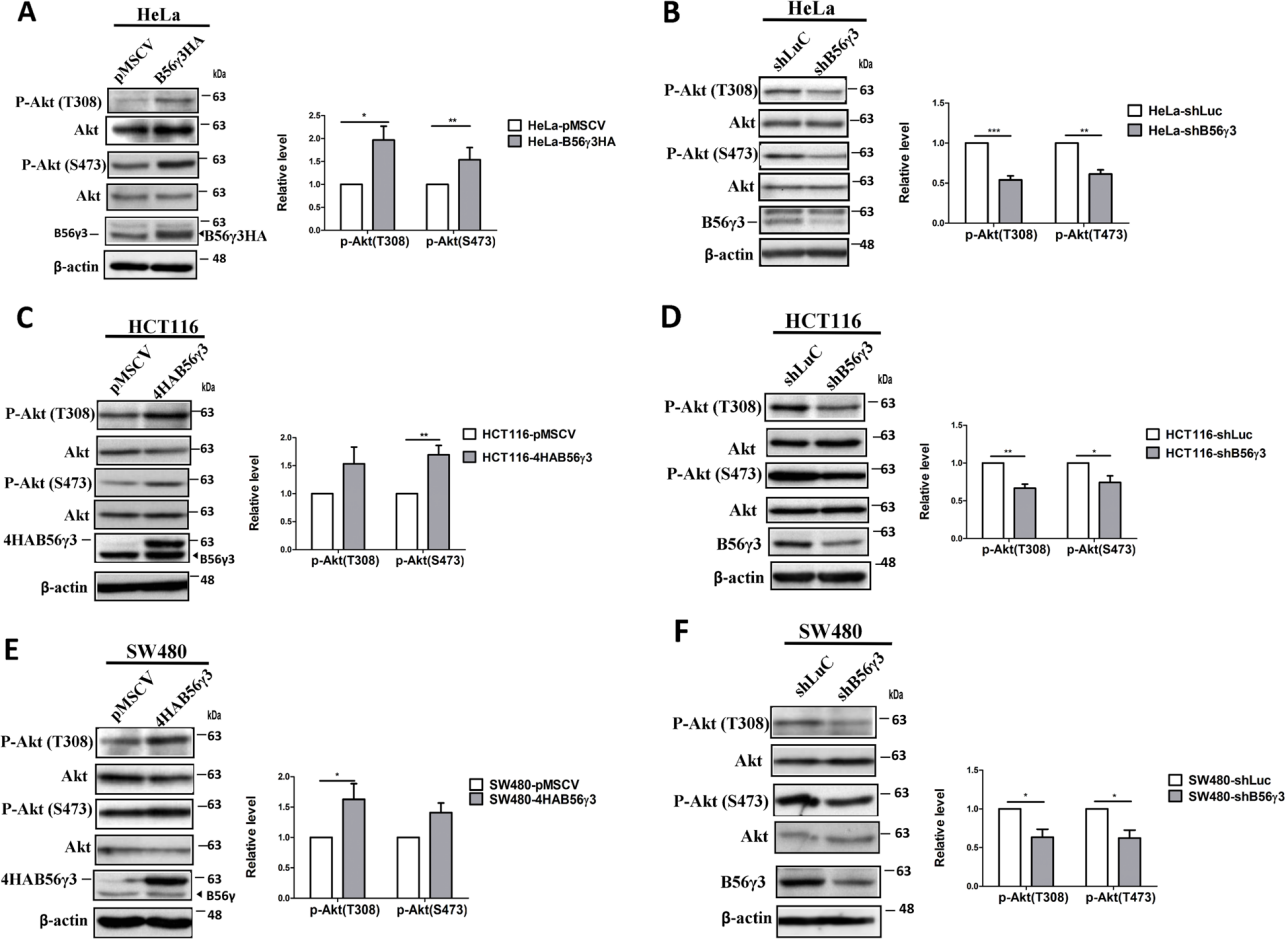
B56γ3 enhances Akt phosphorylation at both Thr308 and Ser473. **A, B **Lysates of HeLa, **C, D **HCT116), and **E, F **SW480 with vector only (pMSCV), stable B56γ3 overexpression (B56γ3HA or 4HAB56γ3) (**A**, **C**, **E**), control shLuc or stable knockdown of B56γ3 expression (shB56γ3) (**B**, **D**, **F**) were harvested at steady state and analyzed by SDS-PAGE and Western blotting with antibodies as indicated. Representative blots of phospho-Akt (p-Akt) at Thr308 and p-Akt at Ser473 from three independent experiments with similar results were shown. The mean relative expression levels of p-Akt at Thr308 (T308) and Ser473 (S473) of three independent experiments with similar results were quantitated and normalized with total Akt by densitometry. The differences were assessed for statistical significance by two-tailed unpaired student’s t test with *p* value (*(< 0.05),**(< 0.01), ***(< 0.001)). (*n* = 3)

B56γ3 has been shown to serve as a key regulatory subunit to provide PP2A with tumor suppressor activity [20–22]. Intriguingly, at steady state, we observed a tendency that B56γ3 overexpression upregulated AKT phosphorylation, whereas B56γ3 knockdown decreased AKT phosphorylation at Ser473 and Thr308, hallmarks of AKT activation, in CRC cell lines HCT116, SW480, and DLD-1 (Fig. 1 and Supplementary Fig. 1). Additionally, to address whether this phenomenon only existed in CRC cells or not, we also investigated other cell types, and we found that B56γ3 positively regulated AKT phosphorylation in HeLa, NIH3T3, and Hep3B cells as well (Fig. 1 and Supplementary Fig. 1). Furthermore, we investigated whether B56γ3 regulates growth factor-stimulated AKT activation. HeLa cells stably expressing control shLuc or shB56γ3 were treated with insulin for 15 min and 30 min and analyzed for the level of phospho-AKT.

**Fig. 2:**
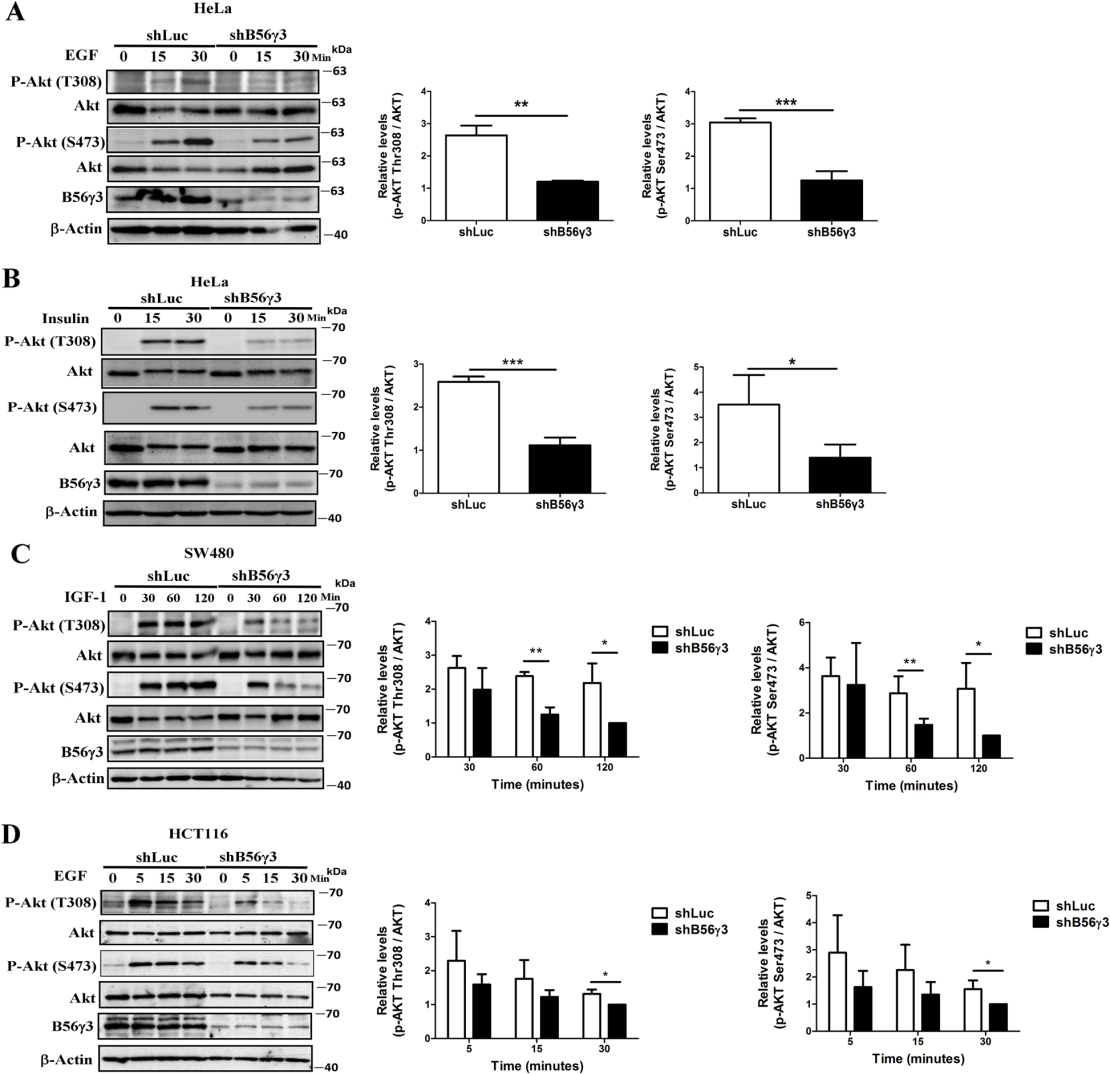
B56γ3 upregulates AKT phosphorylation in response to growth factor stimulation. **A**, **B** Lysates of HeLa cells with shLuc or stable knockdown of B56γ3(shB56γ3) stimulated by EGF (50 ng/ml) or insulin (100 nM), respectively, at the indicated time points, **C** Lysates of SW480 with shLuc or stable knockdown of B56γ3 (shB56γ3) stimulated by IGF-1 (50 ng/ml) at the indicated time points, and **D** Lysates of HCT116 cells with shLuc or stable knockdown of B56γ3(shB56γ3) stimulated by EGF (20 ng/ml) at the indicated time points were analyzed by SDS-PAGE and Western blotting with antibodies as indicated. The mean relative expression levels of p-Akt at Thr308 (T308) and Ser473 (S473) of three independent experiments with similar results were quantitated as described earlier. Graphs show comparisons of the peak levels (**A**, **B**) of phospho-AKT (Thr308 and Ser473) or levels of phospho-Akt (Thr308 and Ser473) at different time points (**C**, **D**) stimulated by growth factors between control shLuc and stable knockdown (shB56γ3). The differences were assessed for statistical significance by Student’s *t* test with p-values (*(< 0.05), **(< 0.01), ***(< 0.001)). (*n* = 3)

As shown in Fig. 2A, the level of EGF-stimulated phospho-Thr308 and -Ser473 of AKT were significantly decreased in HeLa cells stably expressing shB56γ3 compared to that in control cells stably expressing shLuc (Fig. 2A). Moreover, we found that levels of insulin-stimulated phospho-Thr308 and -Ser473 of AKT were significantly decreased in HeLa cells stably expressing shB56γ3 compared to that in control cells with shLuc (Fig. 2B). In addition, SW480 cells stably expressing control shLuc or shB56γ3 were treated with insulin-like growth factor 1(IGF-1) for various time points and results showed that the levels of IGF-1-stimulated phospho-Thr308 and -Ser473 of AKT were significantly decreased in SW480 cells stably expressing shB56γ3 compared to that in control cells with shLuc (Fig. 2C). Moreover, the levels of EGF-stimulated phospho-Thr308 and -Ser473 of AKT were significantly decreased at the later time point (30 min) in HCT116 cells stably expressing shB56γ3 compared to that in control cells with shLuc (Fig. 2D). Collectively, these data suggest that B56γ3 upregulates AKT phosphorylation downstream of insulin, IGF-1 and EGF signaling.

### B56γ3 upregulates AKT phosphorylation by targeting PP2A to attenuate the p70S6K-mediated negative feedback loop regulation

We found that B56γ3 upregulated AKT phosphorylation at both Thr308 and Ser473, which are hallmarks of AKT activation (Figs. 1 and 2). Next, we investigated the mechanism underlying B56γ3-upregulated AKT activation.

**Fig. 3:**
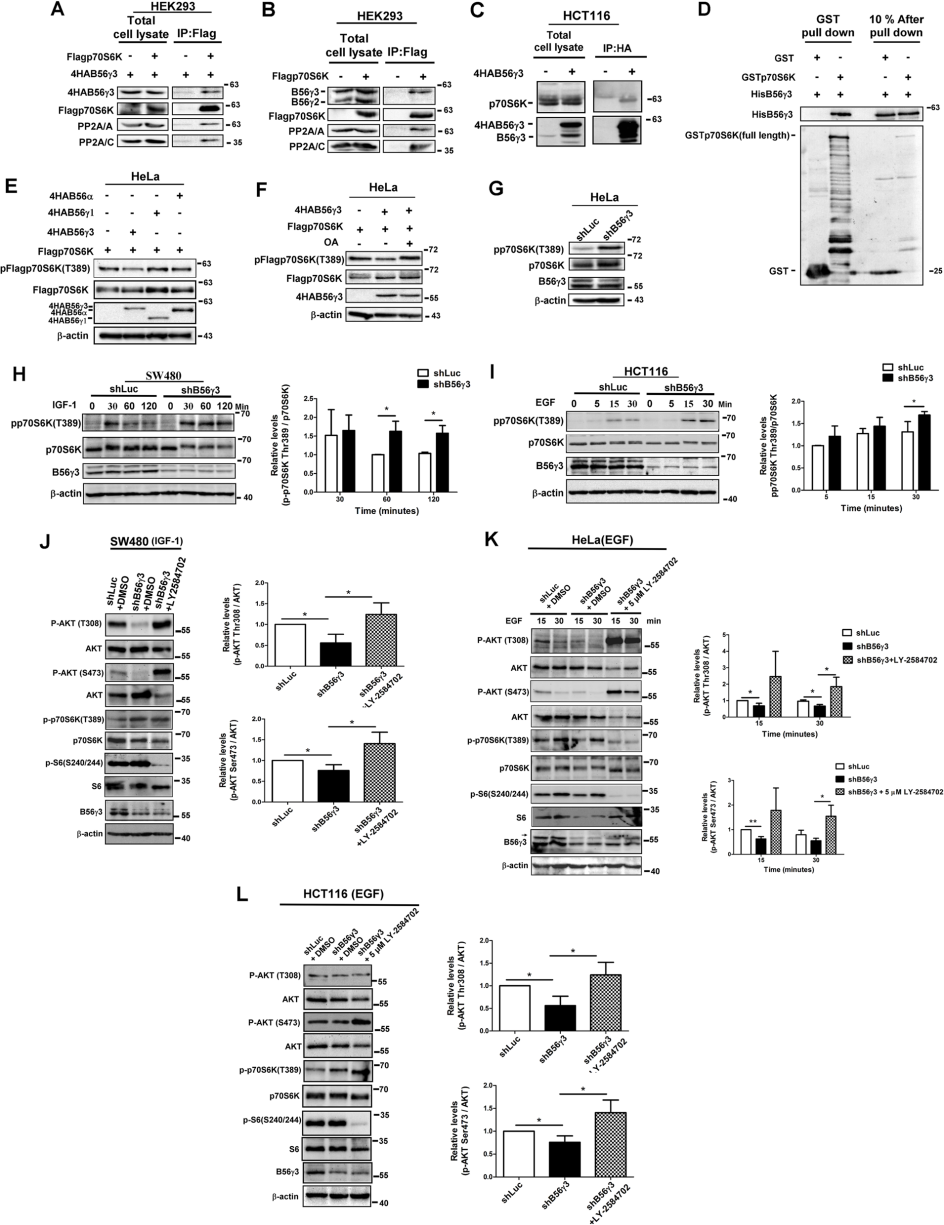
B56γ3 upregulates AKT phosphorylation through targeting PP2A to downregulate p70S6K phosphorylation at Thr389 and attenuate p70S6K-mediated negative feedback loop regulation on growth factor signaling. **A** Lysates of HEK293 cells co-transfected with expression vector encoding 4HA-B56γ3 and Flag-p70S6K or vector only were immunoprecipitated by anti-FLAG-Sepharose, and the immunocomplexes were analyzed by SDS-PAGE and Western blotting with specific antibodies as indicated. **B** Lysates of HEK293 cells transfected with expression vector encoding Flag-p70S6K were immunoprecipitated by anti-FLAG-Sepharose, and the immunocomplexes were analyzed for the presence of endogenous B56γ3. **C** Lysates of HCT116 cells transfected with expression vector encoding 4HA-B56γ3 were immunoprecipitated by anti-HA-Sepharose, and the immunocomplexes were analyzed for the presence of endogenous p70S6K. **D** In vitro pulldown analysis of recombinant GST or GST-p70S6K interacting with recombinant His-B56γ3 using glutathione Sepharose was carried out and GST-pulldowns were then analyzed by SDS-PAGE and Western blotting with antibodies as indicated. Ten percent of mixed recombinant proteins after pulldown analysis were analyzed in parallel, serving as a loading control. **E** Lysates of HeLa cells transfected with expression vector encoding Flag-p70S6K and 4HAB56γ3, 4HAB56γ1, 4HAB56α, or empty vector were analyzed by SDS-PAGE and Western blotting with antibodies as indicated. **F** Lysates of HeLa cells transfected with expression vectors encoding Flag-p70S6K and 4HAB56γ3 treated with or without 500 nM okadaic acid (OA) were analyzed by SDS-PAGE and Western blotting with antibodies as indicated. **G** Lysates of HeLa cells stably expressing shLuc or shB56γ3 were analyzed by SDS-PAGE and Western blotting with antibodies as indicated. **H** Lysates of SW480 cells stably expressing shLuc or shB56γ3 stimulated by IGF-1 (50 ng/ml) at the indicated time points were analyzed as described earlier. **I** Lysates of HCT116 cells stably expressing shLuc or shB56γ3 stimulated by EGF (20 ng/ml) at the indicated time points were analyzed as described earlier. **J** Lysates of SW480 cells with control shLuc or shB56γ3 pretreated with DMSO or 5 μM LY-2584702 (Adooq Bioscience) in serum-free medium, prior to stimulation with IGF-1 (50 ng/ml) for 60 min in the presence or absence of 5 μM LY-2584702, were analyzed as described previously. **K** Lysates of HeLa cells with control shLuc or shB56γ3 pretreated with DMSO or 5 μM LY-2584702 in 0.5% FBS medium prior to stimulation with EGF (50 ng/ml) at the indicated time points in the presence or absence of 5 μM LY-2584702 were analyzed as described previously. **L** Lysates of HCT116 cells with control shLuc or shB56γ3 pretreated with DMSO or 5 μM LY-2584702 in serum-free medium prior to stimulation with EGF (20 ng/ml) for 30 min in the presence or absence of 5 μM LY-2584702 were analyzed as described previously. Representative blots of at least two independent experiments with similar results were shown. The mean relative expression levels of phospho-Akt (Thr308 and Ser473) and phospho-p70S6K (Thr389) of three independent experiments with similar results were quantified as described earlier. The differences were assessed for statistical significance by student’s *t* test with *p* value (*(< 0.05), **(< 0.01), ***(< 0.001)). (*n* = 3)

Given that phosphorylation of Thr308 and Ser473 is selectively catalyzed by PDK1 and mTORC2 [27, 28], and selectively dephosphorylated by PP2A and PHLPP, respectively [23, 29], it is likely that the PP2A holoenzyme containing B56γ3 (PP2A-B56γ3) may indirectly regulate AKT activation by regulating a target which may be simultaneously involved in regulating both phosphorylation of Ser473 and Thr308 of AKT. We hypothesized that p70S6K is a putative target involved in the activation of AKT by PP2A-B56γ3 based on the following findings. PP2A has long been known to negatively regulate p70S6K [15–17]. Knockdown of PPP2R5C, which encodes B56γ1, B56γ2, and B56γ3 subunits, upregulates p70S6K phosphorylation at Thr389 [18]. p70S6K mediates a negative feedback loop regulation to downregulate growth factor signaling and reduce AKT activation [11, 12]. Based on the above findings, we examined whether PP2A-B56γ3 is responsible for dephosphorylation of p70S6K. We co-transfected Flag-p70S6K with 4HA-B56γ3 into HEK293 cells and performed co-immunoprecipitation through pull-down of anti-Flag or anti-HA immunocomplexes. We found that 4HA-B56γ3 was associated with Flag-p70S6K, and endogenous PP2A A and C subunits were present in the anti-FLAG immunocomplexes (Fig. 3A). Reciprocally, when anti-HA immunocomplexes were pulled down, 4HA-B56γ3, Flag-p70S6K, and endogenous PP2A A and C subunits were co-immunoprecipitated (Supplementary Fig. 2A). Furthermore, endogenous B56γ3, but not B56γ2, PP2A A and C subunits were co-immunoprecipitated with Flag-p70S6K (Fig. 3B) and, reciprocally, endogenous p70S6K was co-immunoprecipitated with 4HA-B56γ3 in anti-HA immunocomplexes in HCT116, HEK293 and SW480 cells (Fig. 3C and Supplementary Fig. 2B and C).

Furthermore, in vitro pulldown analysis showed that His-B56γ3 proteins directly associated with GST-p70S6K, but not with GST proteins (Fig. 3D). Next, we examined whether B56γ3, but not other similar B56 subunits, could target PP2A to down-regulate p70S6K phosphorylation. As shown in Fig. 3E, we co-transfected expression vector encoding Flag-p70S6K and 4HA-B56α, 4HA-B56γ3, 4HA-B56γ1HA, or vector only into HeLa cells and examined phospho-Thr389 of Flag-p70S6K. Results showed that 4HA-B56γ3, but not 4HA-B56α or 4HA-B56γ1, significantly reduced phospho-Thr389 levels (Fig. 3E). The dephosphorylation of phospho-Thr389 of Flag-p70S6K by co-expression with 4HA-B56γ3 was restored by okadaic acid (OA), a PP2A selective inhibitor (Fig. 3F). Consistently, the level of phospho-Thr389 of endogenous p70S6K was increased by stably expressing shB56γ3 compared to that of cells expressing shLuc in HeLa cells (Fig. 3G). In addition, we also performed in vitro dephosphorylation assay by incubating various amounts of PP2A-B56γ3 holoenzymes with purified Flag-p70S6K from serum-stimulated lysates, and results showed that PP2A-B56γ3 holoenzymes catalyzed dephosphorylation of phospho-Thr389 in a dose-dependent and OA-sensitive manner, indicating that downregulation of phospho-Thr389 by B56γ3 was carried out by B56γ3 targeting PP2A catalytic activity toward p70S6K (Supplementary Fig. 3). Furthermore, we investigated the role of B56γ3 in regulating growth factor-stimulated p70S6K activation. We found that in NIH3T3 cells, B56γ3 overexpression significantly downregulated insulin-stimulated increases in phospho-Thr389 of p70S6K (Supplementary Fig. 4A). In parallel, B56γ3 overexpression also significantly downregulated amino acid-stimulated phospho-Thr389 of p70S6K in NIH3T3 cells (Supplementary Fig. 4B). On the other hand, B56γ3 knockdown significantly increased IGF-1-stimulated increases in phospho-Thr389 of p70S6K in SW480 cells (Fig. 3H). In addition, B56γ3 knockdown significantly increased EGF-stimulated increases in phospho-Thr389 of p70S6K in HCT116 cells at later time point (Fig. 3I). Given that activation of p70S6K mediates a negative feedback loop regulation to downregulate growth factor signaling and reduce AKT activation [11, 12], we hypothesized that B56γ3 may upregulate AKT activity by mediating PP2A to down-regulate p70S6K activity and subsequently attenuate p70S6K-mediated negative feedback loop regulation. If the hypothesis is correct, constitutively active p70S6K may counteract the B56γ3-mediated upregulation of AKT activation, whereas inhibition of p70S6K activity may restore AKT activation which is downregulated by increased p70S6K activity resulting from B56γ3 knockdown. To verify this hypothesis, NIH 3T3 cells stably expressing vector (pMSCV) only or stably expressing 4HA-B56γ3 were transfected with empty vector, constitutively active (CA) mutant Flag-p70S6K(E389D3E**)**, or kinase-dead (KD) mutant Flag-p70S6K (K100R), and were subsequently stimulated with insulin. Results showed that co-expression of the CA mutant of p70S6K, but not KD mutant of p70S6K, significantly abolished the B56γ3-mediated upregulation of insulin-stimulated phospho-AKT (Supplementary Fig. 5). Next, we investigated whether inhibition of p70S6K activity could restore AKT activation when B56γ3 was silenced. Consistent with our earlier findings (Fig. 3H), levels of IGF-1-stimulated phospho-AKT at both Thr308 and Ser473 were decreased in SW480 cells with B56γ3 knockdown compared to SW480 cells stably expressing shLuc, but inhibition of p70S6K activity with LY-2584702, a selective ATP competitive inhibitor of p70S6K, restored IGF-1-stimulated AKT phosphorylation in SW480 cells with B56γ3 knockdown (Fig. 3J). Treatment with LY-2584702 did not reduce phosphorylation at Thr389 but reduced the activity of p70S6K, causing reduced levels of phospho-S6 (Thr240/244), an immediate downstream substrate of p70S6K (Fig. 3J). Similarly, treatment with LY-2584702 restored levels of EGF-stimulated AKT phosphorylation at both Thr308 and Ser473 in both HeLa and HCT116 cells with B56γ3 knockdown (Fig. 3K and L). Taken together, these data suggest that B56γ3 upregulates AKT activation by targeting PP2A to downregulate p70S6K activation, resulting in attenuating the p70S6K-mediated negative feedback loop regulation.

### B56γ3 regulates EMT in CRC cells

**Fig. 4:**
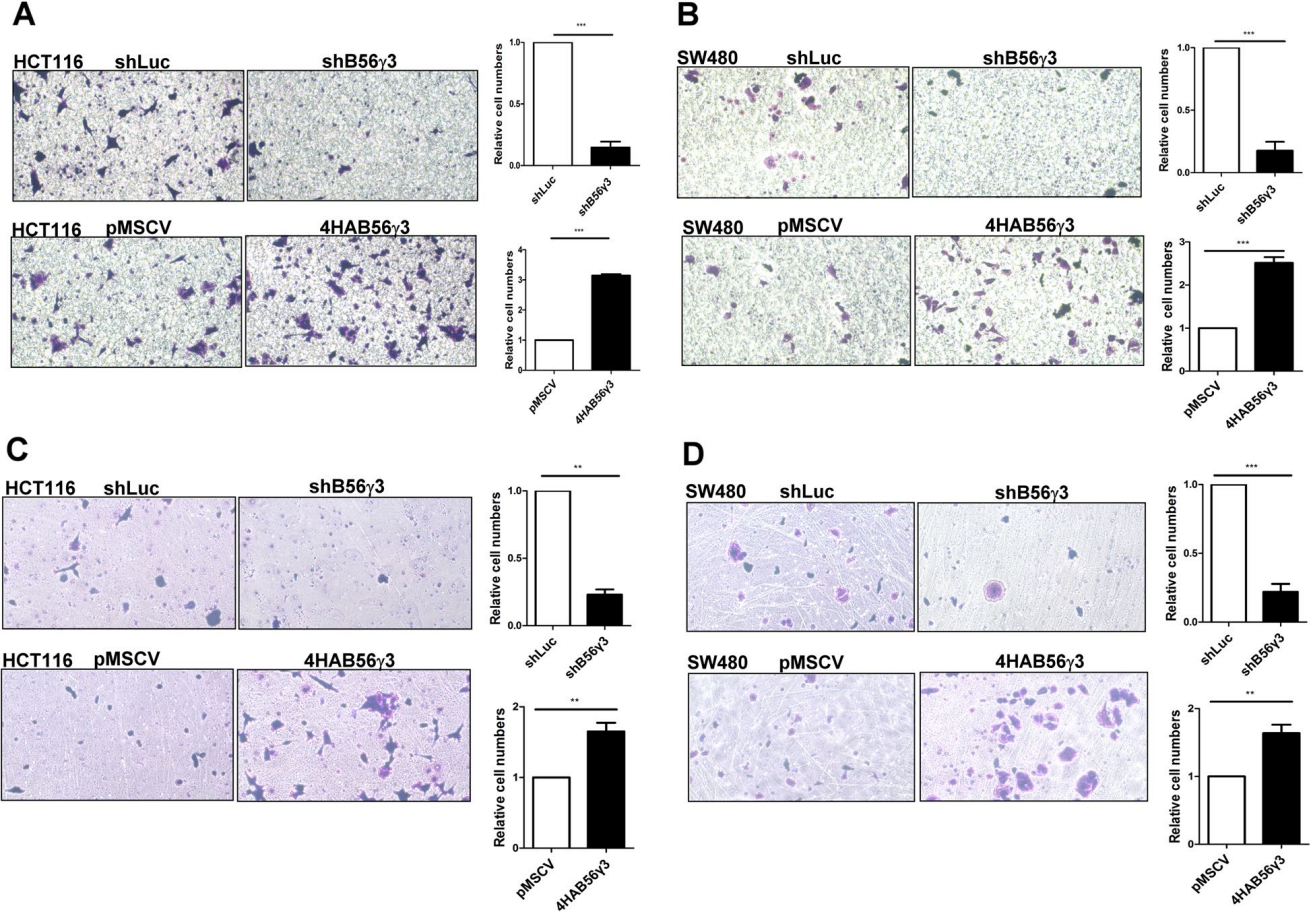
B56γ3 facilitates motility and invasion of CRC cells. **A** HCT116 cells **B** SW480 cells with control vector (pMSCV or shLuc), stable overexpression of B56γ3 (4HAB56γ3), or stable knockdown of B56γ3 (shB56γ3) were seeded onto the upper transwell chamber, and migrated cells were stained with crystal violet and counted 24 h after seeding. **C** HCT116 or **D** SW480 cells with the control vector (pMSCV or shLuc), stable overexpression of B56γ3 (4HAB56γ3), or stable knockdown of B56γ3 (shB56γ3) were seeded onto the upper chamber precoated with Matrigel for Transwell invasion analyses, and migrated cells were stained with crystal violet and counted 24 h after seeding. Representative images from at least three independent experiments are shown. Quantitation of migrated cells in different experimental groups was performed using ImageJ, and differences in relative cell numbers were assessed for statistical significance by Student’s *t* test with *p* value (*(< 0.05), **(< 0.01), ***(< 0.001)). (*n* = 3)

We showed that B56γ3 promotes AKT phosphorylation, which is an oncogenic hallmark of various types of cancer and a master regulator of EMT through different mechanisms [30]. Since we showed that B56γ3 upregulated AKT activity in HCT116 and SW480 cells both of which are CRC cell lines (Figs. 1, 2 and 3), we investigated whether B56γ3 plays a role in EMT in CRC cells. We performed Transwell migration assay in HCT116 and SW480 cells to assess the role of B56γ3 in the motility of CRC cells. Results showed that the number of both HCT116 and SW480 cells with B56γ3 knockdown migrating through the filter was much lower than that of cells with shLuc (Fig. 4A and B). On the other hand, B56γ3 overexpression promoted cell migration as compared to that of cells with vector only (Fig. 4A and B). In addition, the in vitro scratch wound-healing migration assay showed that B56γ3 played a positive role in regulating cell migration in SW480 cells (Supplementary Fig. 6). Since an increase in invasion ability is also a hallmark of EMT, we further investigated the impact of B56γ3 on the invasion ability of CRC cells. Results of in vitro Transwell invasion assay showed that both SW480 and HCT116 cells with B56γ3 knockdown showed reduced number of cells invading into the lower chamber as compared to that of cells with shLuc, whereas B56γ3 overexpression increased cell invasion into the lower chamber in both SW480 and HCT116 cells as compared to cells with vector only (Fig. 4C and D). Furthermore, PPP2R5C which encodes B56γ family members including B56γ3 was knocked out by Crispr/Cas 9 approach. Our data showed that expression of B56γ3 was abolished in PPP2R5C knockout clones of HCT116 cells as compared to that of parental cells (Supplementary Fig. 7). Knockout clones of HCT116 cells showed markedly reduced spindle-shaped and scattering morphology, but increased epithelial morphology and had reduced capability of migration and invasion as compared to parental cells (Supplementary Fig. 7). Together, these data demonstrate that B56γ3 promotes EMT of CRC cells.

### B56γ3 upregulates the level of SNAIL and hallmarks of EMT through enhancing AKT activity

**Fig. 5:**
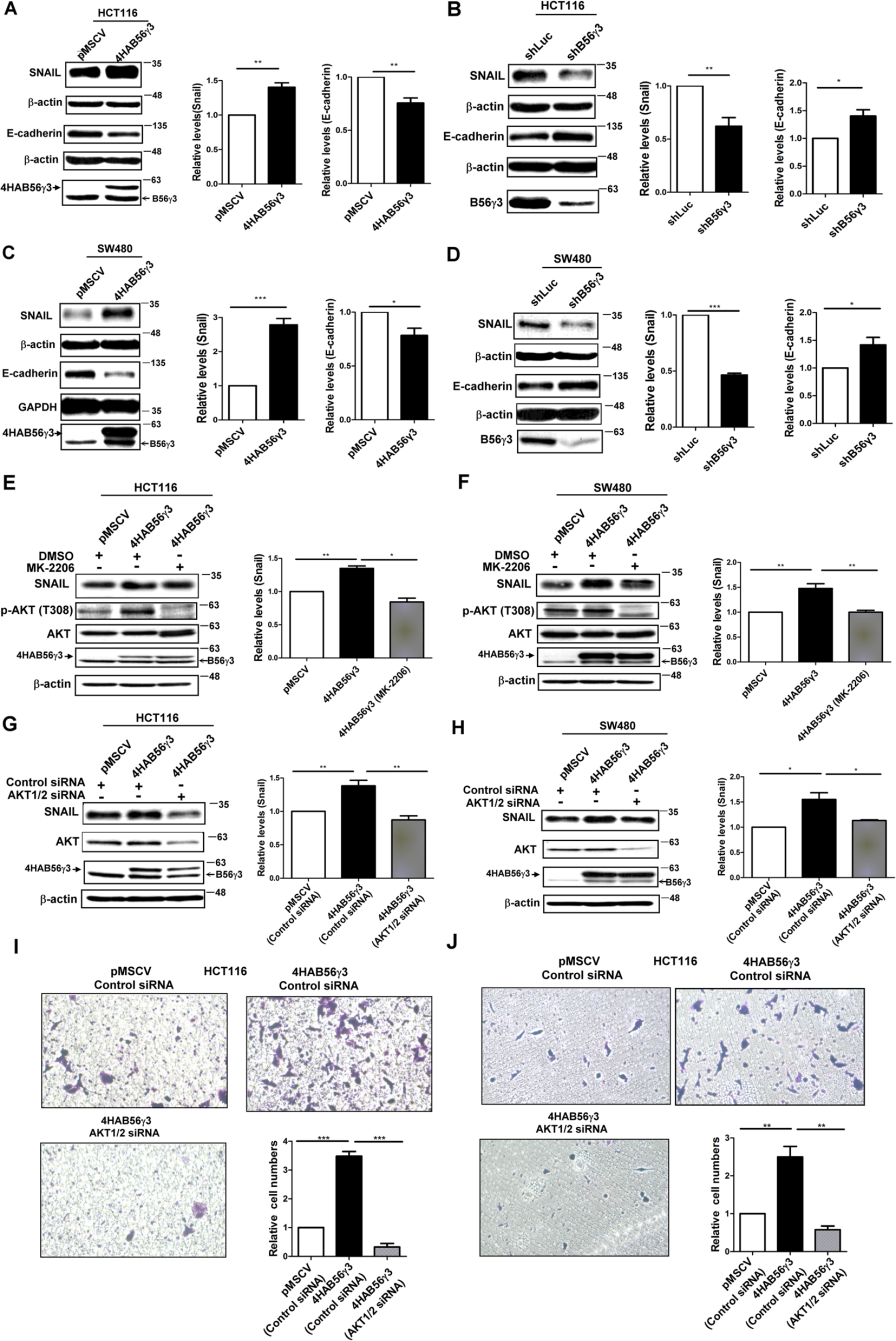
B56γ3 upregulates SNAIL and EMT hallmarks through AKT activity. **A**, **B** Lysates of HCT116 cells or **C**, **D** lysates of SW480 cells with the control vector (pMSCV or shLuc), stable overexpression of B56γ3 (4HAB56γ3), or stable knockdown of B56γ3 (shB56γ3) were analyzed by SDS-PAGE and Western blotting with specific antibodies as indicated. **E** Lysates of HCT116 or **F** lysates of SW480 cells with vector only (pMSCV) or stable overexpression of B56γ3 (4HAB56γ3) treated with DMSO or MK-2206(10 μM, Enzo Life Sciences) for 24 h were analyzed as described previously. **G** Lysates of HCT116 or **H** lysates of SW480 cells with vector only (pMSCV) or stable overexpression of B56γ3 (4HAB56γ3) transiently transfected with control siRNA or AKT1/2 siRNA (120 nM) were analyzed as described previously. The relative expression levels of SNAIL and E-cadherin were quantified by densitometry and normalized to that of β-actin. **I** HCT116 cells with vector only (pMSCV) or stable overexpression of B56γ3 (4HAB56γ3) transiently transfected with control siRNA or AKT1/2 siRNA (120 nM) were subjected to Transwell migration assay or **J** Transwell invasion assay as described earlier. The differences were assessed for statistical significance by Student’s t-test with *p*-values (* (< 0.05), ** (< 0.01), ***(< 0.001))

To investigate the underlying mechanism of B56γ3-promoted EMT, we investigated the role of B56γ3 on regulating the expression of the key EMT transcription factor SNAIL whose level is known to be regulated by AKT [30]. The results showed that SNAIL was increased by B56γ3 overexpression, and was reduced by B56γ3 knockdown in HCT116 and SW480 cells (Fig. 5A-D)*.* In agreement with the role of B56γ3 in regulating SNAIL, the level of E-cadherin, an epithelial marker negatively regulated by SNAIL, was reduced by B56γ3 overexpression, whereas B56γ3 knockdown increased the level of E-cadherin in both HCT116 and SW480 cells (Fig. 5A-D). To verify the role of AKT in B56γ3-mediated increases of SNAIL levels, we treated CRC cells overexpressing B56γ3 with an AKT-specific inhibitor, MK-2206, to inhibit AKT activity.

Our results showed that treatment with MK-2206 significantly decreased AKT phosphorylation and inhibited the B56γ3-mediated increased levels of SNAIL in both HCT116 and SW480 cells (Fig. 5F and G). In addition, when CRC cells were treated with AKT-specific siRNAs to knock down AKT1/2 expression, results showed that reduced expression of AKT1/2 by siRNAs blocked B56γ3-mediated increases in SNAIL levels, cell motility and invasion of HCT116 and SW480 cells (Fig. 5G-J and Supplementary Fig. 8). Moreover, we found that the level of phospho-GSK3β at Ser 9, which is a target site of AKT and crucial for regulating SNAIL levels, were reduced when B56γ3 expression was silenced in CRC cells (Supplementary Fig. 9). Taken together, these data indicate that B56γ3 upregulates SNAIL levels involved in EMT by upregulating AKT activity.

### B56γ3 reduces sensitivity of CRC cells to 5-FU

**Fig. 6:**
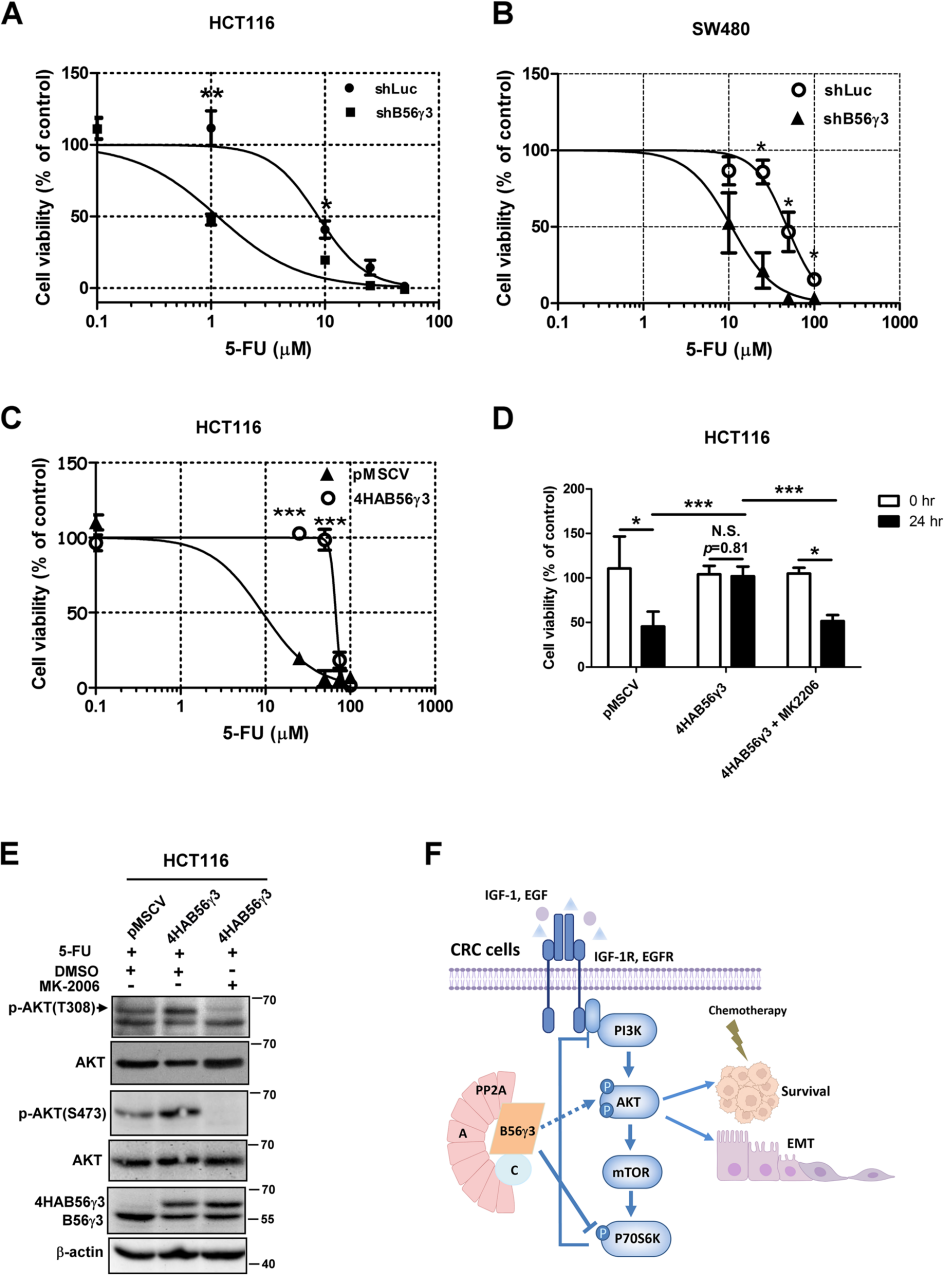
B56γ3 reduces the sensitivity of CRC cells to 5-FU. **A** HCT116 and **B** SW480 cells with control vector (shLuc) or stable knockdown of B56γ3 (shB56γ3) were treated with 0, 25, 50, 75, and 100 μM 5-FU for 72 h, and cell viability was measured using the Presto Blue viability assay according to the manufacturer’s protocol. **C** HCT116 cells with vector only (pMSCV) or stably overexpressing B56γ3 (4HAB56γ3) were treated with 5-FU, and cell viability was measured as described previously. The IC50 of 5-FU was analyzed using GraphPad Prism version 5 for Windows. **D** HCT116 cells with vector (pMSCV) or stable overexpression of B56γ3 (4HAB56γ3) were treated with 5-FU (50 μM) or 5-FU (50 μM) and MK-2206 (50 μM) for 24 h, and cell viability was measured as described previously. **E** Lysates of HCT116 cells with vector only (pMSCV) or B56γ3 overexpression (4HAB56γ3) treated with 5-FU or 5-FU and MK2206 for 24 h were analyzed by SDS-PAGE and Western blotting with the indicated antibodies. **F** The PP2A-B56γ3 holoenzyme upregulates AKT activation to promote EMT and the potential of drug resistance by downregulating p70S6K activity and attenuating the p70S6K-mediated negative feedback loop regulation of growth factor-stimulated PI3K/AKT activation. The dashed line indicates that the PP2A-B56γ3 holoenzyme indirectly activates AKT activity

Given that B56γ3 upregulates AKT activity and that hyperactive PI3K/AKT signaling has been linked to multidrug resistance in a variety of cancer types [31, 32], next, we investigated whether B56γ3 played a role in regulating cancer cell survival upon chemotherapeutic drug treatment. We treated CRC cells with 5-Fluorouracil (5-FU) at various doses and the viability of CRC cells treated with 5-FU was assessed. We found that knockdown of B56γ3 expression reduced the viability of HCT116 and SW480 cells compared with cells stably expressing control shLuc (Fig. 6A and B). The IC50 of HCT116 cells with B56γ3 knockdown (IC50: 1.146 μM) under 5-FU treatment was eight times lower than that of HCT116 cells with shLuc (IC50: 8.72 μM) (Fig. 6A). In addition, the IC50 of SW480 cells with B56γ3 knockdown under 5-FU treatment was also significantly reduced (IC50: 10.76 μM) as compared to that of SW480 cells with shLuc (IC50: 48.16 μM) (Fig. 6B). On the other hand, B56γ3 overexpression markedly increased survival of HCT116 cells by 5-FU treatment (Fig. 6C). The IC50 of HCT116 cells with B56γ3 overexpression treated with 5-FU was 67.33 μM, whereas the IC50 of HCT116 cells stably expressing empty vector was 9.257 μM (Fig. 6C). Next, we verified the role of AKT in regulating B56γ3-promoted survival upon 5-FU treatment by treating B56γ3-overexpressing HCT116 cells with the selective AKT inhibitor MK-2206. Results showed that HCT116 cells with B56γ3 overexpression maintained nearly 100% viability when nearly 50% of HCT116 cells with the vector only died by 5-FU treatment at 24 h. However, the B56γ3 overexpression-mediated increases in viability was abolished by co-treatment with MK-2206 (Fig. 6D). In agreement with results of the viability analysis, treatment with MK-2206 abolished B56γ3 overexpression-enhanced AKT phosphorylation in HCT116 cells treated with 5-FU (Fig. 6E). Together, our results indicate that B56γ3 reduced the sensitivity and increased the viability of CRC cells treated with 5-FU by increased AKT activity.

### The level of B56γ3 is positively correlated with the level of phospho-AKT in CRC tissue specimens and is inversely correlated with survival of CRC patients

**Fig. 7:**
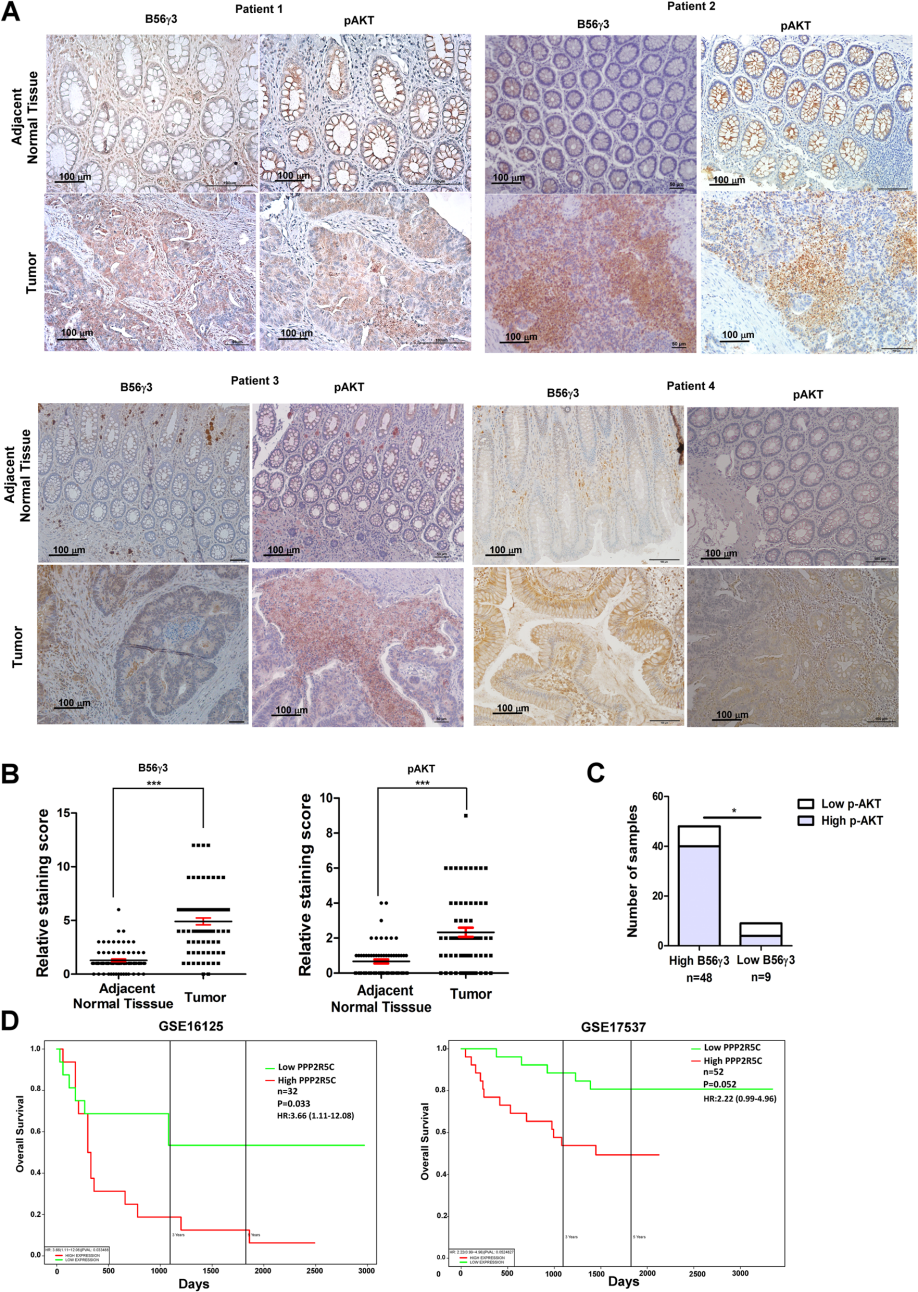
B56γ3 expression was upregulated in CRC and correlated with AKT activation and poor prognosis. **A** Representative micrographs of IHC analysis of B56γ3 and phosphorylation of AKT (p-AKT, Ser473) in tumor parts and normal tissue counterparts of 57 human colon cancer specimens are shown. Micrographs shown are four paired tissue specimens with higher IHC scores of both B56γ3 and p-AKT in tumor parts compared to that in normal tissue counterparts. Micrographs are shown at magnification 200X. The scale bar is 50–100 μm. **B** Scatter plots shown are quantitative analysis of scores of staining of expression of B56γ3 (Left) and p-AKT (Right) in both tumor and non-tumor parts of 57 paired human colon cancer specimens. **C** Data shown are concordance analysis of IHC scores of B56γ3 and p-AKT in tumor tissues. **D** Kaplan–Meier survival analysis of two cohorts (GSE16125 and GSE17537) of CRC patients with PPP2R5C (B56γ) high or PPP2R5C (B56γ) low expression. *P* values were calculated using two-tailed unpaired Student’s t-test with *p* value (* (< 0.05), ** (< 0.01), ***(< 0.001))

We showed that B56γ3 upregulated the phosphorylation of AKT and EMT in human CRC cell lines (Figs. 1, 4 and 5). Next, we investigated whether there was any correlation between the levels of B56γ3 and phospho-AKT in human CRC specimens. IHC analysis of 57 pairs of human CRC tissue and adjacent normal tissue specimens revealed that both B56γ3 and phospho-AKT were highly expressed in tumor tissues compared to their adjacent normal tissues in the majority of the specimens (Fig. 7A and B). In addition, 40 of 57 pairs of specimens had overexpressed B56γ3 and phospho-AKT in the tumor tissues, indicating that expression of B56γ3 was positively associated with that of phospho-AKT in a subset of human CRC specimens (Fig. 7C). Next, we analyzed the relationship between mRNA expression of PPP2R5C which encodes different B56γ isoforms and the survival rates of patients with CRC using datasets of CRC in public databases. We found that CRC patients with higher PPP2R5C mRNA levels in tumors showed poorer overall survival rates than those with lower PPP2R5C mRNA levels in tumors in the two CRC cohorts (Fig. 7D). In addition to CRC, we found that higher PPP2R5C mRNA levels in tumors showed poorer overall survival rates in several other types of cancer, including adrenocortical adenocarcinoma, uveal melanoma, esophageal carcinoma, and kidney carcinoma (Supplementary Fig. 10). Taken together, these data suggest that the levels of B56γ3 were positively correlated with those of phospho-AKT and that high B56γ3 levels were associated with poor prognosis in a subset of CRC patients.

## Discussion

Here, we report that the B56γ3 regulatory subunit of PP2A upregulated AKT activation through downregulating p70S6K-mediated negative feedback loop regulation (Figs. 1, 2 and 3). Moreover, B56γ3 promoted EMT and decreased the sensitivity of CRC cells to chemotherapy by upregulating AKT activation (Fig. 6). Importantly, we found that high expression of B56γ3 is associated with poor prognosis of a subset of CRC patients (Fig. 7).

Phosphorylation of Akt at two key residues, Thr308 and Ser473, was required for its full activation [27, 28]. We previously showed that the B55α-containing PP2A selectively catalyzes dephosphorylation of AKT at Thr308 and downregulates AKT activity [23]. Here, we observed that B56γ3 promoted AKT phosphorylation at both Thr308 and Ser473 not only in CRC cells, but also in several other cell types (Fig. 1 and Supplementary Fig. 1). We characterized the molecular mechanism underlying B56γ3 upregulated AKT phosphorylation and revealed that upregulation of AKT phosphorylation by B56γ3 is an indirect consequence of B56γ3 targeting PP2A dephosphorylation and inactivation of p70S6K, which has been shown to mediate a negative feedback loop in downregulating PI3k/AKT signaling downstream of growth factors (Fig. 6F). Since p70S6K is a downstream signal transducer of PI3k/AKT signaling and mediates a negative feedback loop in downregulating PI3k/AKT signaling, we found that the amplitude of phosphorylation of AKT and p70S6K was more significantly modulated by B56γ3 knockdown than by B56γ3 overexpression (Figs. 1, 2 and 3 and Supplementary Fig. 1 and 4), which is consistent with a previous report showed that the complex signaling network topology by kinases or phosphatases is modulated in a protein abundance-dependent manner [33]. On the other hand, the B56γ-containing PP2A was shown to dephosphorylate and inactivate ERK [34], which may upregulate the mTORC1/ p70S6K signaling through inactivating tuberous sclerosis complex (TSC) 2 [35]; therefore, we cannot rule out the possibility that the B56γ3-containing PP2A can also indirectly downregulate p70S6K through ERK inactivation. Given that AMPK was shown to downregulate the mTOR/p70S6K signaling module through the phosphorylation of raptor or TSC2 or indirectly through protein phosphatases [36, 37], the possibility that cross-talk between the B56γ3-containing PP2A and AMPK results in inactivation of p70S6K cannot be ruled out. To further address the kinetics of upregulating AKT phosphorylation by B56γ3, we re-expressed B56γ3 in a PPP2R5C knockout clone of HCT116 cells using tetracycline-inducible approach, and our results showed that increased phosphorylation of AKT at both Thr308 and Ser473 occurred at 48 h after induced expression of B56γ3 but not in control PPP2R5C knockout cells treated with doxycycline alone (Supplementary Fig. 11). However, at 48 h after induction of B56γ3 expression, phosphorylation of p70S6K at Thr389 was reduced in both parental and the B56γ3 re-expressing knockout clone of HCT116 cells, suggesting that increased cell density may downregulate phosphorylation of p70S6K and hinder the effect of B56γ3 expression (Supplementary Fig. 11).

Our finding of upregulated AKT activation in CRC cells by the PP2A-B56γ3 holoenzyme was similar to the finding that cancer cells treated with mTOR inhibitors, such as rapamycin and Torin, exhibited hyperactivation of PI3K/AKT signaling by attenuating mTOR/p70S6K-mediated negative feedback loops [38]. The mechanism of upregulated AKT activity by the PP2A-B56γ3 holoenzyme is also similar to that of MAPK phosphatases in regulating MEK1/2, which are regulated by ERK1/2-mediated negative feedback loop downstream of growth factor stimulation [39]. It was shown that overexpression of MAPK phosphatases (MKPs/DUSPs) caused sustained MEK1/2 activation via suppressing the negative feedback regulation from ERK1/2 to MEK1/2 [33, 40]. The expression of MKPs was found to be positively associated with cancer progression and overexpressed MKPs increased the malignancy of cancers with a hyperactivated MAPK-ERK pathway [39, 41]. Consistently, we demonstrated that both B56γ3 and phospho-AKT were overexpressed and positively correlated in tumors in a subset of CRC specimens (Fig. 7). Moreover, we found that high B56γ3 mRNA levels were associated with poor prognosis in two cohorts of CRC patients and several other types of cancer (Fig. 7 and Supplementary Fig. 10).

B56γ3 has been recognized to act as a tumor suppressor regulatory subunit of PP2A by targeting the PP2A holoenzyme to regulate the level and activity of key cell cycle regulators to control cell proliferation and tumorigenicity [20–22]. Nevertheless, our data demonstrated that B56γ3 promoted EMT through upregulating AKT activity in CRC cells, suggesting that B56γ3 may act as a tumor suppressor or oncogene in a context-dependent manner such as tumors harboring aberrant B56γ3 overexpression and mutations causing hyperactivated PI3K/AKT (Fig. 4). In addition, some studies have shown that EMT is associated with cell cycle arrest [42, 43]. It is conceivable that B56γ3 overexpression not only promotes EMT by upregulating AKT activity but also further augments EMT by regulating cell cycle arrest through regulating p53 and p27Kip1. EMT is linked to the properties of cancer stem cells (CSCs), which have been shown to have increased resistance to conventional cancer therapies [44], and in agreement with this notion, we showed that B56γ3 overexpression reduced the sensitivity of CRC cells to 5-FU treatment by upregulating AKT activity (Fig. 6). Since ATM was shown to phosphorylate B56γ3 and increase the level of the B56γ3-containing PP2A after DNA damage [45], it is also likely that ATM activation by 5-FU treatment may further increase the B56γ3-containing PP2A to enhance AKT activity to reduce drug sensitivity in CRC cells with stable B56γ3 overexpression. Moreover, the interaction of B56γ3 and p70S6K may serve as a therapeutic target for the treatment of CRC and other types of cancer when B56γ3 is overly expressed under hyperactivated PI3K/AKT signaling. Altogether, our findings demonstrate that the PP2A-B56γ3 holoenzyme may switch from a tumor suppressor to an oncogene in a cell context-dependent manner.

## Conclusion

PP2A-B56γ3 has been recognized to function as a tumor suppressor. Herein, our results reveal that PP2A-B56γ3 can play an oncogenic role in CRC. PP2A-B56γ3 upregulates EMT and reduces the sensitivity to the chemotherapeutic drug 5-FU through upregulating AKT activity. Mechanistically, PP2A-B56γ3 upregulates AKT activity by suppressing the p70S6K-mediated negative feedback loop. Moreover, the level of B56γ3 is positively correlated with the level of phospho-AKT in tumor tissue specimens of CRC and is inversely correlated with survival of patients with CRC. Our finding suggests that the PP2A-B56γ3/p70S6K signaling module may serve as a therapeutic target to control tumor progression and drug resistance in CRC with high expression of both B56γ3 and phospho-AKT.

## Supplementary Information

**Additional file 1: Supplemental Table 1.** List of antibodies applied in this study.

**Additional file 2: Supplementary Fig. 1.** B56γ3 enhances Akt phosphorylation at both Thr308 and Ser473. **Supplementary Fig. 2.** The PP2A-B56γ3 holoenzyme physically interacts with p70S6K. **Supplementary Fig. 3.** The PP2A-B56γ3 holoenzyme catalyzes dephosphorylation of phospho-p70S6Kin vitro. **Supplementary Fig. 4.** B56γ3 downregulates p70S6K activation in response to insulin stimulation and supply of amino acids in NIH3T3 cells. **Supplementary Fig. 5.** B56γ3 upregulates AKT activation through downregulating p70S6K-mediated negative feedback loop regulation on growth factor signaling. **Supplementary Fig. 6.** B56 3 promotes wound healing migration in CRC cells. **Supplementary Fig. 7.** PPP2R5C knockout clones of HCT116 cells showed reduced mesenchymal phenotypes. **Supplementary Fig. 8.** B56 3 promotes EMT hallmarks in CRC cells through enhancing AKT activation. **Supplementary Fig. 9.** Knockdown of B56γ3 decreases phosphorylation of GSK3β at Ser9 in CRC cells. **Supplementary Fig. 10.** High expression of B56γ3 associates with poor prognosis in adrenocortical carcinoma, esophageal carcinoma, uveal melanoma, and kidney chromophobe carcinoma. **Supplementary Fig. 11.** The upregulation of AKT phosphorylation by reexpression of B56γ3 in a PPP2R5C knockout clone of HCT116 cells occurred at 48 h after B56γ3 re-expression.

## Data Availability

Besides data of CRC cancer datasets were obtained through PROGgeneV2 (http://www.progtools.net/gene), all data generated or analyzed during this study are included in this published article.

## Abbreviations

CRC: Colorectal Cancer
EMT: Epithelial-Mesenchymal Transition
GEO: Gene Expression Omnibus
5-FU: 5-Fluorouracil
mTOR: Mammalian target of rapamycin
OA: Okadaic Acid
PP2A: Protein Phosphatase 2A
PP2A-B56γ3: The B56γ3 regulatory subunit-containing PP2A holoenzyme
shLuc: ShRNA against luciferase
shB56γ3: ShRNA against B56γ3
TCGA: The Cancer Genome Atlas
TSC: Tuberous sclerosis complex
