# Supplementary material for: The B56γ3-containing protein phosphatase 2A attenuates p70S6K-mediated negative feedback loop to enhance AKT-facilitated epithelial-mesenchymal transition in colorectal cancer
Source: Cell Commun Signal. 2023 Jul 10;21:172. doi: 10.1186/s12964-023-01182-5 (PMC10332018; doi:10.1186/s12964-023-01182-5)
Supplement: Supplementary file 2 — Additional file 1: Supplemental Table 1. List of antibodies applied in this study. [file 12964_2023_1182_MOESM1_ESM.pdf]

Supplemental Table 1 List of antibodies applied in this study

Antibody	Company	Catalog	Species	Application
**Primary antibodies**				
Anti-AKT	BD	610860	Mouse	WB
Anti-PP2Acα	BD	610555	Mouse	WB
Anti-phospho-AKT (Thr308)	Cell Signaling Technology.	#13038	Rabbit	WB
Anti-phospho-AKT (Ser473)	Cell Signaling Technology.	#4060	Rabbit	WB, IHC
Anti-phospho-p70S6K	Cell Signaling Technology.	#9206	Mouse	WB
Anti-p70S6K	Cell Signaling Technology.	#9202	Rabbit	WB
Anti-HA	Cell Signaling Technology.	(C29F4) #3724	Rabbit	WB
Anti-α-tubulin	Millipore	#05-829	Mouse	WB
Anti-B56γ3	Santa Cruz	E-6, sc-374380	Mouse	WB, IHC
Anti-PP2A/A	Santa Cruz	C-20, sc-6112	Goat	WB
Anti-p70S6K	Sigma-Aldrich	SAB2500736	Goat	WB
Anti-FLAG	Sigma-Aldrich	F3165	Mouse	WB
Anti-β-actin	Sigma-Aldrich	A5441	Mouse	WB
**Secondary antibodies**				
HRP-conjugated anti-mouse IgG	Jackson ImmunoResearch	315-035-008		WB
HRP-conjugated anti-rabbit IgG	Jackson ImmunoResearch	115-035-003		WB
HRP-conjugated anti-goat IgG	Santa Cruz	sc-2350/sc-2354		WB
HRP-conjugated anti-mouse IgG, light chain specific	Jackson ImmunoResearch	115-035-174		WB
