# Supplementary material for: The B56γ3-containing protein phosphatase 2A attenuates p70S6K-mediated negative feedback loop to enhance AKT-facilitated epithelial-mesenchymal transition in colorectal cancer
Source: Cell Commun Signal. 2023 Jul 10;21:172. doi: 10.1186/s12964-023-01182-5 (PMC10332018; doi:10.1186/s12964-023-01182-5)
Supplement: Supplementary file 3 — Additional file 2: Supplementary Fig. 1. B56γ3 enhances Akt phosphorylation at both Thr308 and Ser473. Supplementary Fig. 2. The PP2A-B56γ3 holoenzyme physically interacts with p70S6K. Supplementary Fig. 3. The PP2A-B56γ3 holoenzyme catalyzes dephosphorylation of phospho-p70S6Kin vitro. Supplementary Fig. 4. B56γ3 downregulates p70S6K activation in response to insulin stimulation and supply of amino acids in NIH3T3 cells. Supplementary Fig. 5. B56γ3 upregulates AKT activation through downregulating p70S6K-mediated negative feedback loop regulation on growth factor signaling. Supplementary Fig. 6. B56 3 promotes wound healing migration in CRC cells. Supplementary Fig. 7. PPP2R5C knockout clones of HCT116 cells showed reduced mesenchymal phenotypes. Supplementary Fig. 8. B56 3 promotes EMT hallmarks in CRC cells through enhancing AKT activation. Supplementary Fig. 9. Knockdown of B56γ3 decreases phosphorylation of GSK3β at Ser9 in CRC cells. Supplementary Fig. 10. High expression of B56γ3 associates with poor prognosis in adrenocortical carcinoma, esophageal carcinoma, uveal melanoma, and kidney chromophobe carcinoma. Supplementary Fig. 11. The upregulation of AKT phosphorylation by reexpression of B56γ3 in a PPP2R5C knockout clone of HCT116 cells occurred at 48 h after B56γ3 re-expression. [file 12964_2023_1182_MOESM2_ESM.pdf]

Supplementary information

The B56γ3-containing protein phosphatase 2A attenuates p70S6K-mediated negative feedback loop to enhance AKT-facilitated epithelial-mesenchymal transition in colorectal cancer

Kai-Ching Hsiao[1]*, Siou-Ying Ruan[1]*, Shih-Min Chen[1]*, Tai-Yu Lai[2], Ren-Hao Chan[3], Yan-Ming Zhang[1], Chien-An Chu[4], Hung-Chi Cheng[5], Hung-Wen Tsai[4], Yi-Fang Tu[6], Brian K. Law[7], Ting-Tsung Chang[8], Nan-Haw Chow[4], and Chi-Wu Chiang[1, 2 ^]

[1]Institute of Molecular Medicine, [2]Institute of Basic Medical Sciences, College of Medicine, National Cheng Kung University, Tainan, Taiwan, ROC, [3] Department of Surgery, National Cheng Kung University Hospital, College of Medicine, National Cheng Kung University, Tainan, Taiwan, ROC, [4] Department of Pathology, National Cheng Kung University Hospital, College of Medicine, National Cheng Kung University, Tainan, Taiwan, ROC, [5]Department of Biochemistry and Molecular Biology, College of Medicine, National Cheng Kung University, Tainan, Taiwan, ROC, [6]Department of Pediatrics, National Cheng Kung University Hospital, College of Medicine, National Cheng Kung University, Tainan, Taiwan, ROC, [7]Department of Pharmacology and Therapeutics and the UF-Health Cancer Center, University of Florida, Gainesville, Florida 32610, USA, [8]Department of Internal Medicine, National Cheng Kung University Hospital, College of Medicine, National Cheng Kung University, Tainan, Taiwan, ROC

Running title: PP2A-B56γ3 promotes EMT by upregulating AKT activity in CRC

Key words: AKT/colorectal cancer/EMT/PP2A-B56γ3/p70S6K

*These authors contributed equally.
^Corresponding author: Chi-Wu Chiang, Institute of Molecular Medicine, College of Medicine, National Cheng Kung University, No.1 University Rd., 701 Tainan, Taiwan Tel: (6) 235-3535 (ext. 3637); Fax: (6) 209-5845; E-mail: chiangcw@mail.ncku.edu.tw

Table of contents

Supplementary figures S1-S11 **Page 2-11**

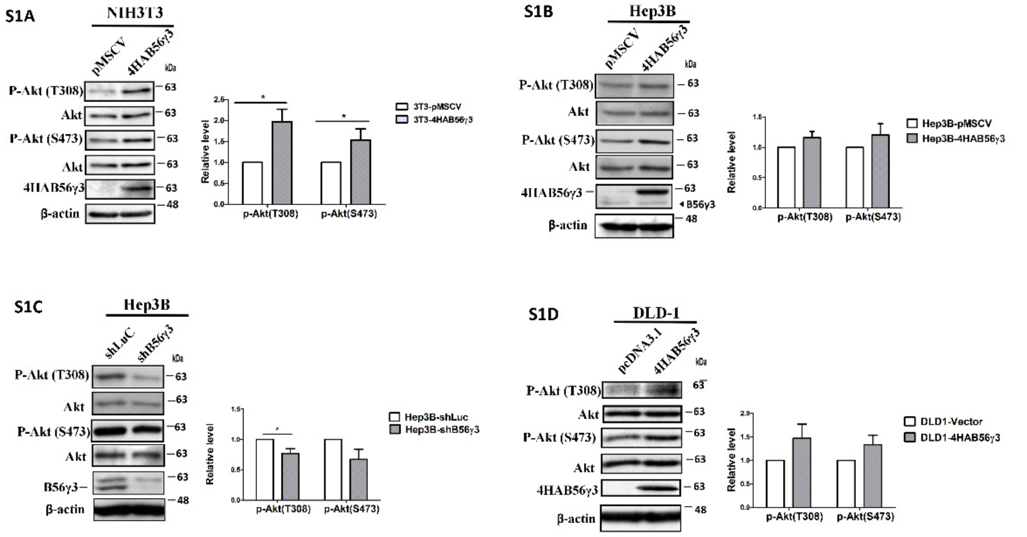

Supplementary Figure 1. B56γ3 enhances Akt phosphorylation at both Thr308 and Ser473. Lysates of NIH3T3 (**A**), Hep3B (**B, C**), and DLD-1 (**D**) with vector only (pMSCV, pcDNA3.1), B56γ3 overexpression (4HAB56γ3), control shLuc, or stable knockdown of B56γ3 expression (shB56γ3) were harvested at steady state and analyzed by SDS-PAGE and Western blotting with antibodies as indicated. Representative blots of phospho-Akt (p-Akt) at Thr308 (Left panel) and p-Akt at Ser473 (Right panel) from three independent experiments with similar results were shown. The mean relative expression levels of p-Akt at Thr308 (T308) and Ser473 (S473) of three independent experiments with similar results were quantitated and normalized with total Akt by densitometry. The differences were assessed for statistical significance by two-tailed unpaired student's t test with p value (*(<0.05),**(<0.01), ***(<0.001)). (n=3)

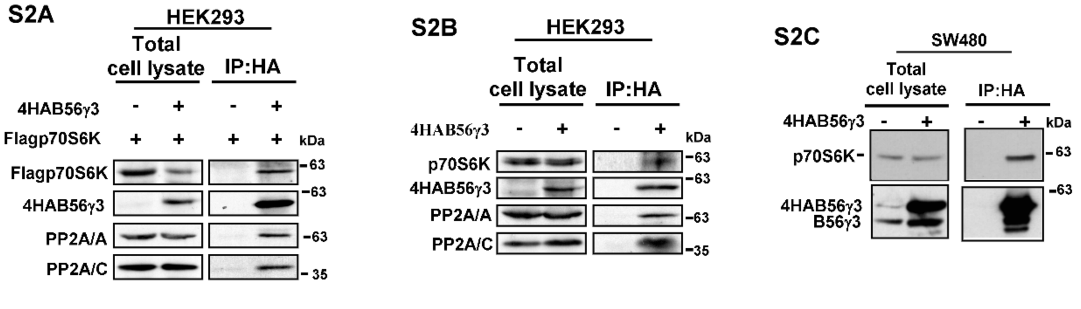

Supplementary Figure 2. The PP2A-B56γ3 holoenzyme physically interacts with p70S6K. (**A**). Lysates of HEK293 cells co-transfected with expression vector encoding Flag-p70S6K and 4HA-B56γ3 or vector only were immunoprecipitated by anti-HA-Sepharose and the immunocomplexes were analyzed as described above. (**B**). Lysates

of HEK293 cells transfected with expression vector encoding 4HA-B56γ3 were immunoprecipitated by anti-HA-Sepharose, and the immunocomplexes were analyzed for the presence of endogenous p70S6K. (**C**). Lysates of SW480 cells transfected with expression vector encoding 4HA-B56γ3 were immunoprecipitated by anti-HA-Sepharose, and the immunocomplexes were analyzed for the presence of endogenous p70S6K.

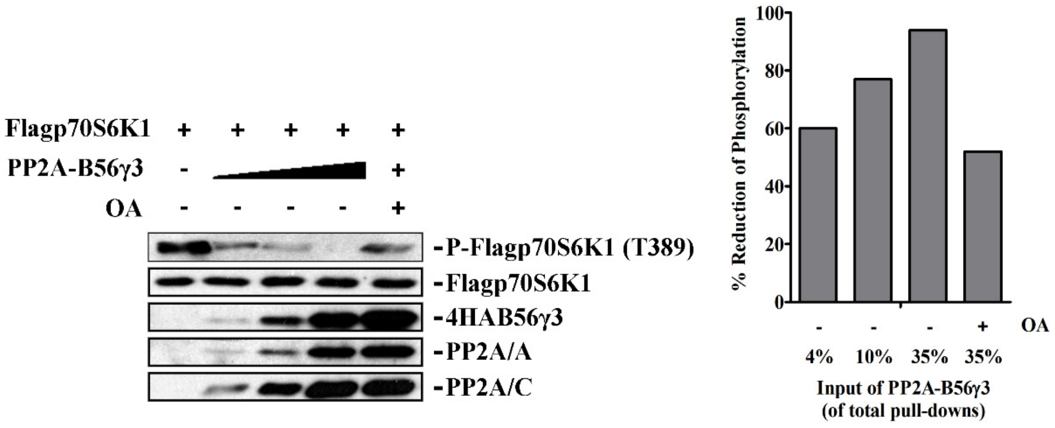

Supplementary Figure 3. The PP2A-B56γ3 holoenzyme catalyzes dephosphorylation of phospho-p70S6K (Thr389) *in vitro*. In vitro dephosphorylation reactions of purified phospho-Flagp70S6K in the absence or presence of various amounts of the purified PP2A-B56γ3 holoenzyme complexes with or without 100 nM OA were carried out at 37℃ for 30 minutes. Expression levels of phospho-Flag-p70S6K (Thr389), Flag-p70S6K1, 4HAB56γ3, and PP2A A and C subunits were detected by Western blotting with antibodies specific for phospho-p70S6K (Thr389), FLAG-tag, HA-tag, PP2A/A, and PP2A/C. The levels of p70S6K phosphorylation were quantified by densitometry and normalized with total p70S6K. Levels of p70S6K phosphorylation in control reactions with no addition of PP2A-B56γ3 complexes were set as 100 %. Data expressed as percentages of reduction of

phospho-p70S6K in individual reactions in the presence of PP2A-B56γ3 complexes with or without OA. Data shown are from one representative experiment of two independent experiments with similar results.

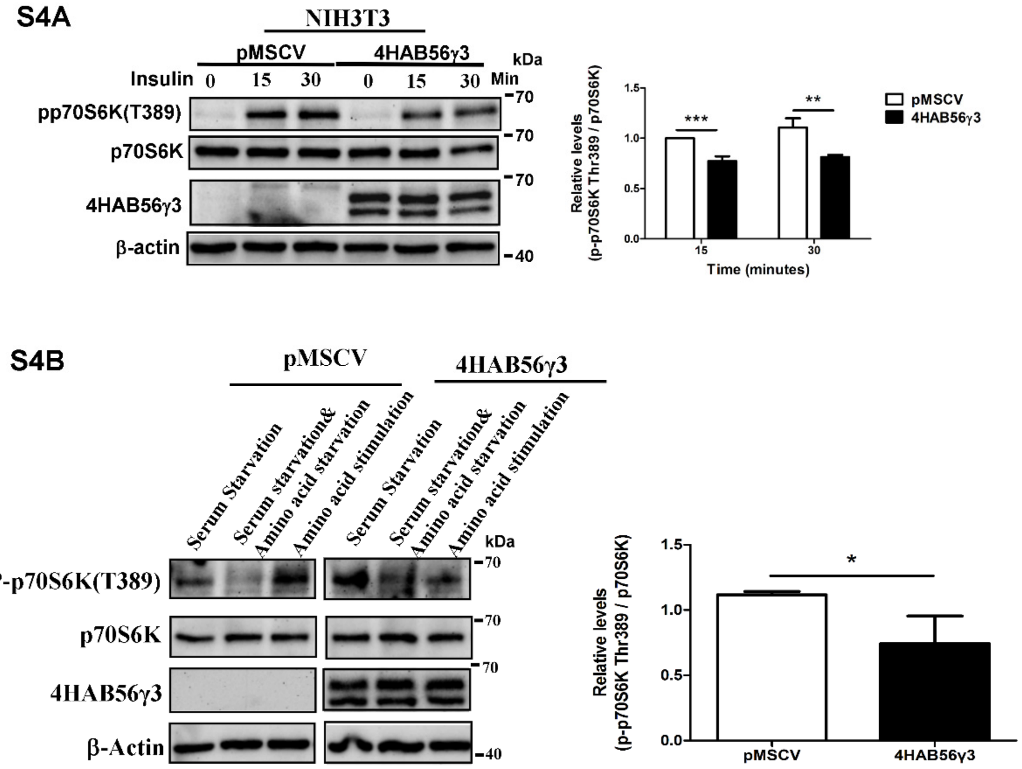

Supplementary Figure 4. B56γ3 downregulates p70S6K activation in response to insulin stimulation and supply of amino acids in NIH3T3 cells. (**A**), Lysates of NIH3T3 cells with pMSCV vector only or stable 4HAB56γ3 overexpression were stimulated with insulin (100 nM) at indicated time points or (**B**) pre-incubated in EBSS 30 min and followed by stimulation by 1x amino acid for 30 min after serum starvation for 16 hours, were analyzed by SDS-PAGE and Western blotting with specific antibodies as indicated. Graphs shown are comparisons of the relative levels of p-p70S6K (Thr389) stimulated by insulin or amino acids between cells with control

vector (pMSCV) and cells with stable B56γ3 overexpression (4HAB56γ3). Data shown are from one representative experiment of three independent experiments with similar results. Levels of p70S6K phosphorylation in cells with control vector (pMSCV) stimulated with insulin (at 15 min) or amino acids were set as 1. The differences were assessed for statistical significance by student's t test with p value (*(<0.05),**(<0.01), ***(<0.001)).(n=3)

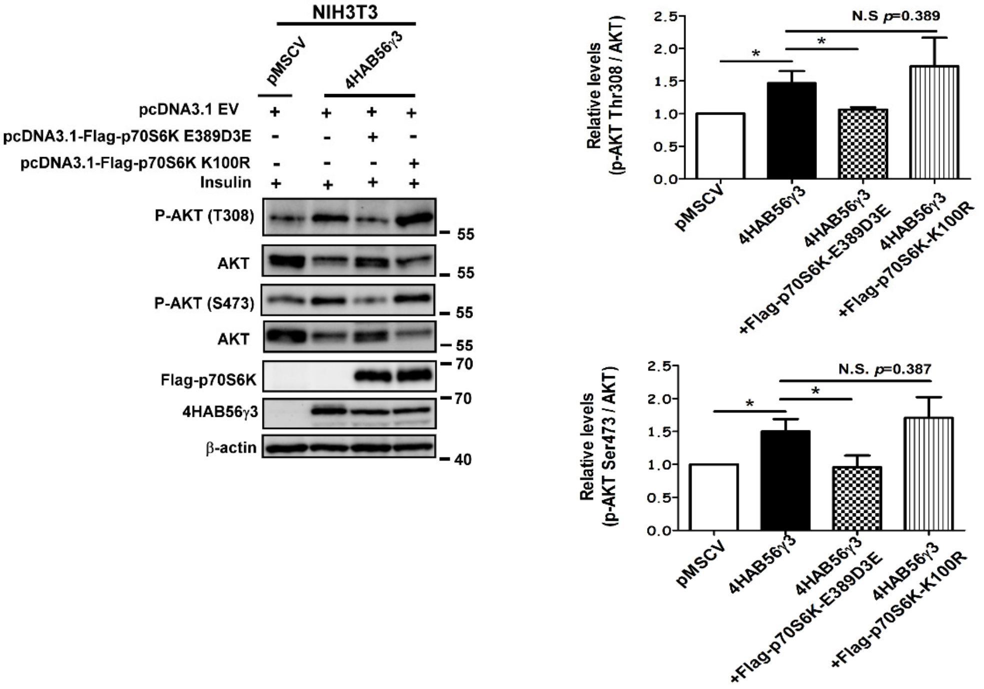

Supplementary Figure 5. B56γ3 upregulates AKT activation through downregulating p70S6K-mediated negative feedback loop regulation on growth factor signaling. Lysates of NIH3T3 cells with vector only (pMSCV) or stable B56γ3 overexpression (4HAB56γ3) transfected with empty vector pcDNA3.1 (EV), Flag-p70S6K E389E3D, or Fag-p70S6K K100R, followed by treatment with insulin (100 nM) for 30 min, were analyzed by SDS-PAGE and western blotting with the indicated antibodies. Data shown are from one representative experiment of three independent experiments with similar results. The relative expression levels of phospho-Akt

(Thr308 and Ser473) were quantified and normalized by densitometry as described previously. Levels of AKT phosphorylation in control cells with control vector (pMSCV) were set as 1. The differences were assessed for statistical significance by student's t test with p value (*(<0.05),**(<0.01), ***(<0.001)).(n=3)

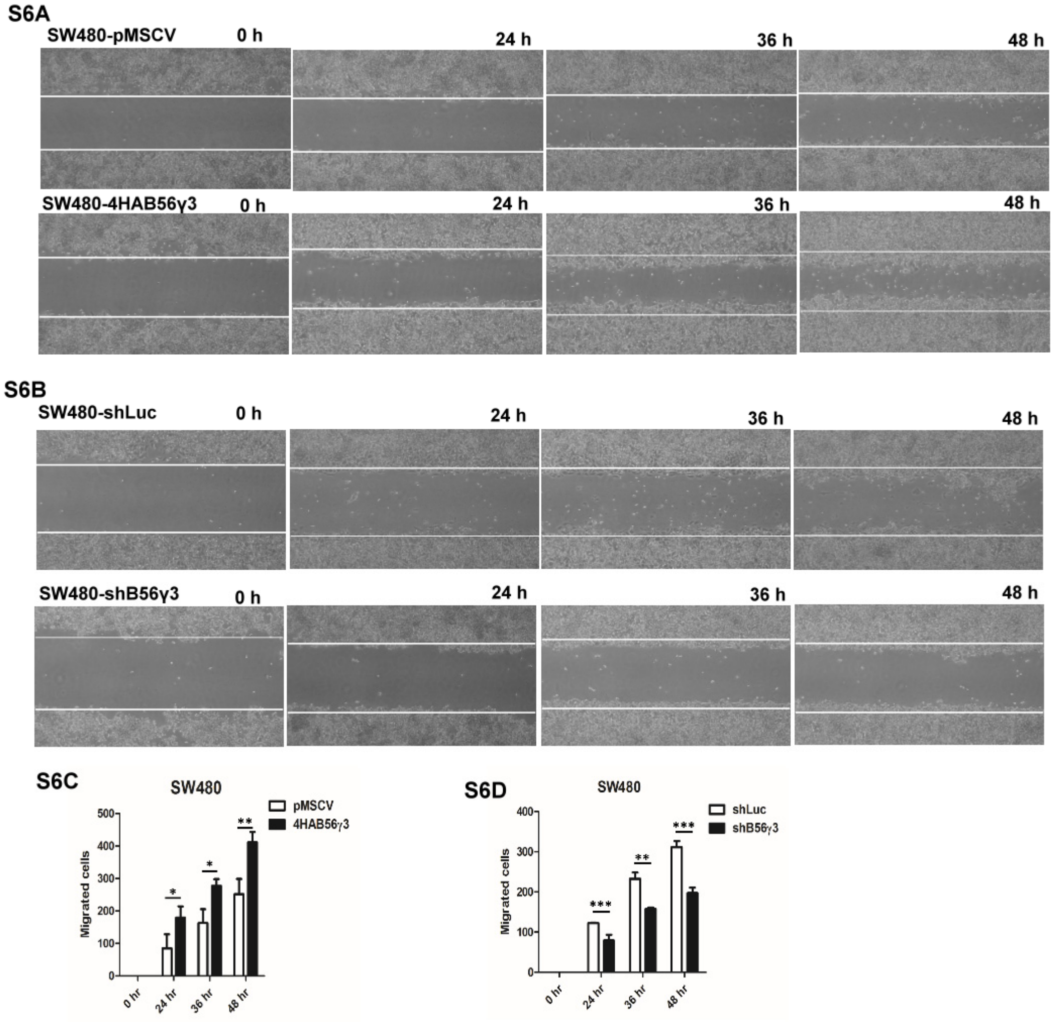

Supplementary Figure 6. B56γ3 promotes wound healing migration in CRC cells. (A, B) SW480 cells with vector only (pMSCV), control shLuc, stable overexpression of B56γ3 (4HAB56γ3), or stable knockdown of B56γ3 (shB56γ3) were seeded onto the 6-cm dish and grown to 90% confluency. Monolayers of cells were subjected to scratch wound healing migration assay. Images were taken at 0, 24, 36, and 48 hours (hr) after

wounds recovery. **(C, D)** Graphs shown are quantitation analysis for migrated cells in A and B using Image J**.** The differences were assessed for statistical significance by student's t test with p value (*(<0.05), **(<0.01), ***(<0.001)). (n=3)

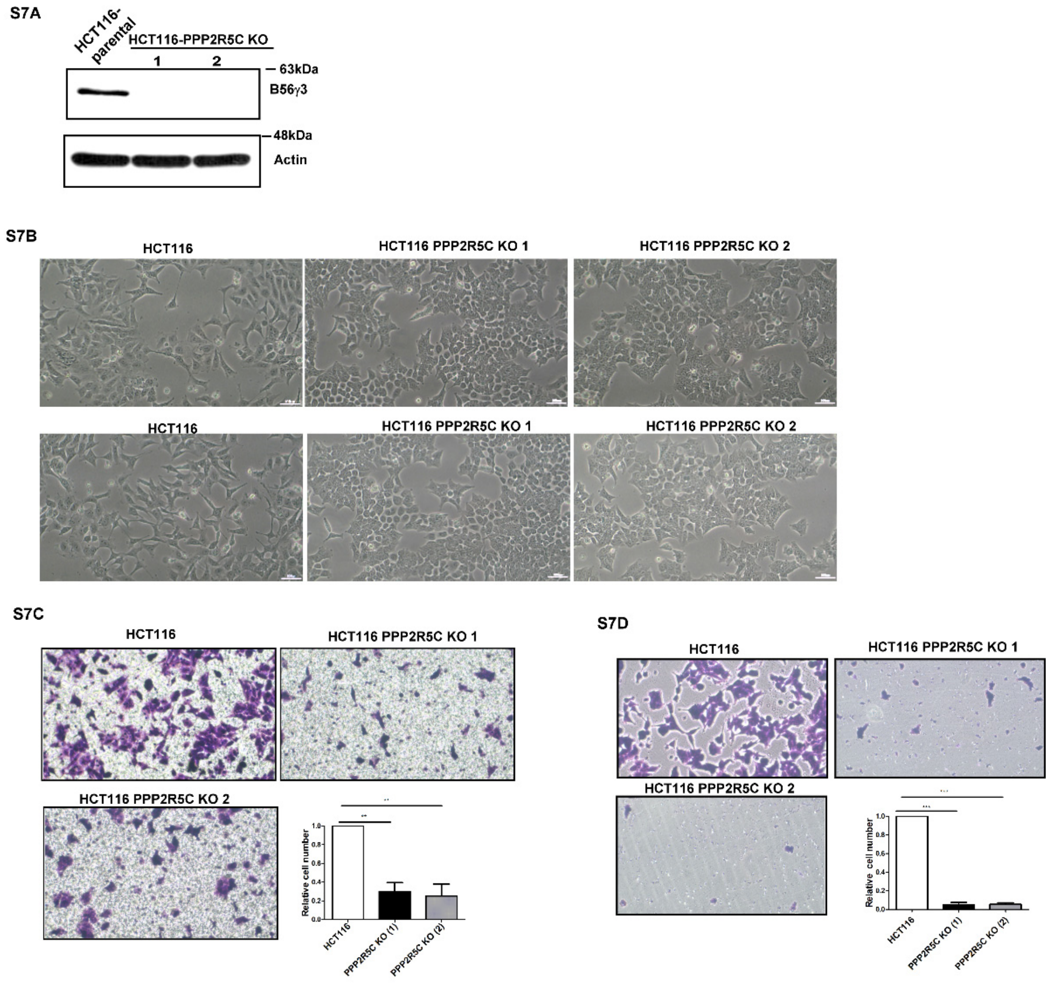

Supplementary Figure 7. PPP2R5C knockout clones of HCT116 cells showed reduced mesenchymal phenotypes. (**A**). Expression of the B56γ3 protein in two PPP2R5C knockout (KO) clones of HCT116 cells was analyzed by SDS-PAGE and western blotting using antibodies as indicated. (**B**). Images of the parental HCT116 cells and two PPP2R5C knockout (KO) clones of HCT116 cells were photographed with a

digital camera at 100X magnification at 14-16 h after seeding. (**C**). Transwell migration analyses of the parental and two PPP2R5C KO clones of HCT116 cells was performed as described previously. (**D**). Transwell invasion analyses of the parental and two PPP2R5C KO clones of HCT116 cells was performed as described previously.

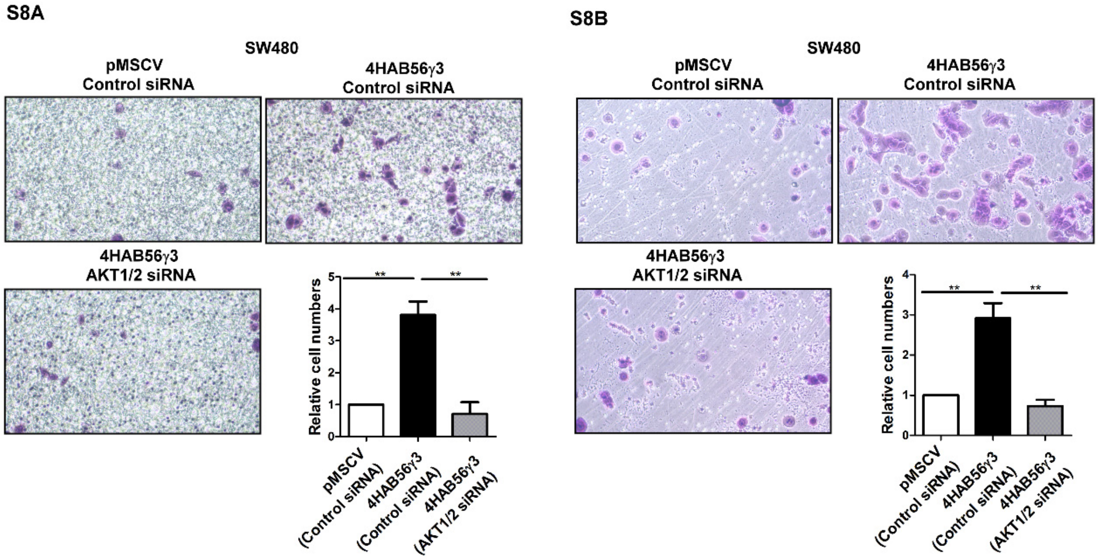

Supplementary Figure 8. B56γ3 promotes EMT hallmarks in CRC cells through enhancing AKT activation. (A, B) SW480 cells with vector only (pMSCV) or stable overexpression of B56γ3 (4HAB56γ3) transiently transfected with control siRNA or AKT1/2 siRNA (120 nM) were subjected to transwell migration analysis (A) or transwell invasion analysis (B) as described earlier. Representative images from at least three independent experiments are shown. Quantitation of migrated cells in different experimental groups was performed using Image J, and differences in relative cell numbers were assessed for statistical significance by Student's *t* test with p value (*(<0.05), **(<0.01), ***(<0.001)). (n=3)

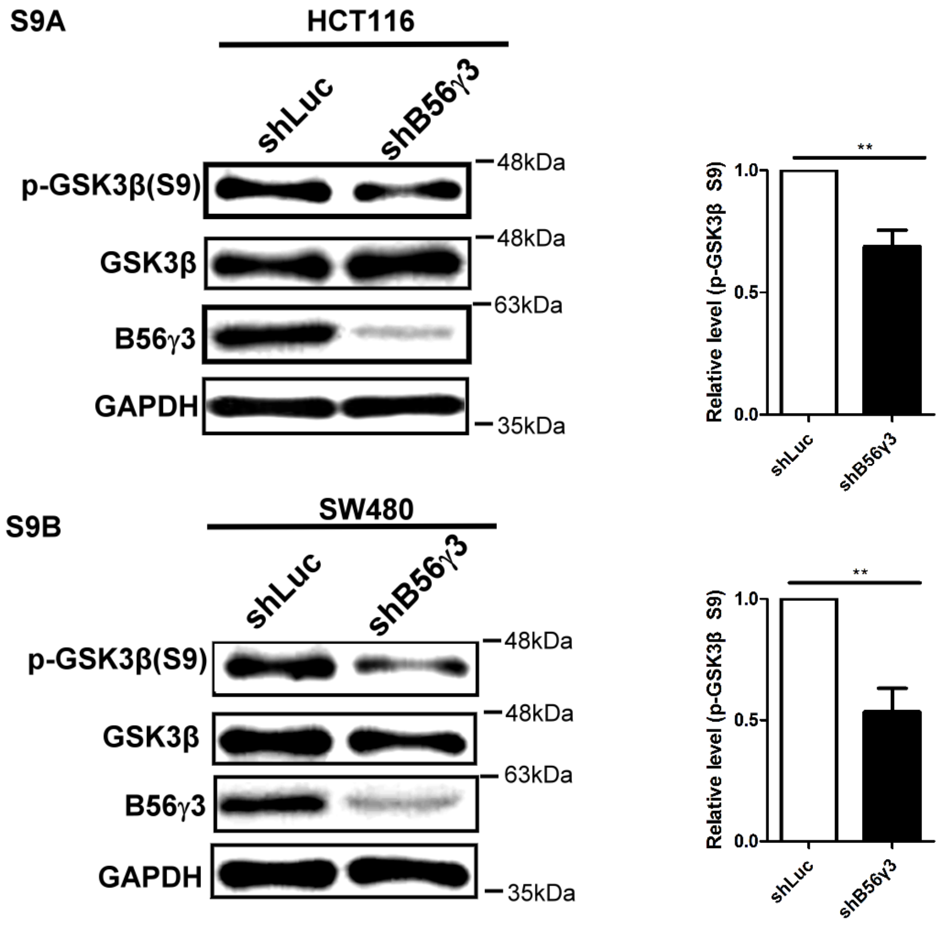

Supplementary Figure 9. Knockdown of B56γ3 decreases phosphorylation of GSK3β at Ser9 in CRC cells. Lysates of HCT116 (A) or SW480 (B) cells with control shLuc or stable knockdown of B56γ3 (shB56γ3) were analyzed by Western blotting with specific antibodies as indicated. The relative expression levels of phospho-GSK3β(S9) were quantified and normalized with total GSK3β. The data shown are expressed as –fold expression level over that of control shLuc, which was set as 1. The differences were assessed for statistical significance by student's t test with p value (*(<0.05), **(<0.01), ***(<0.001)). (n=3)

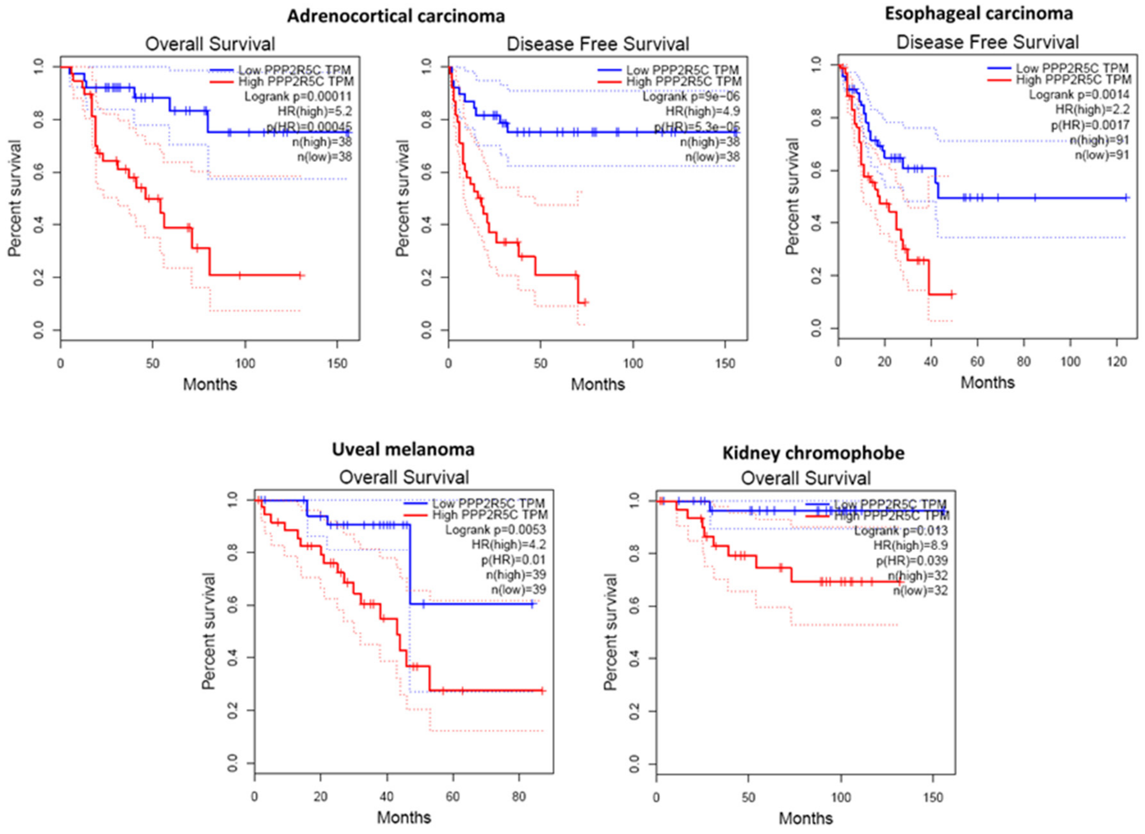

Supplementary Figure 10. High expression of B56γ3 associates with poor prognosis in adrenocortical carcinoma, esophageal carcinoma, uveal melanoma, and kidney chromophobe carcinoma. Graphs shown are Kaplan-Meier survival analysis of overall or disease-free survival of patients with PPP2R5C (B56γ) high or PPP2R5C (B56γ) low expression of TCGA datasets using GEPIA database[1].

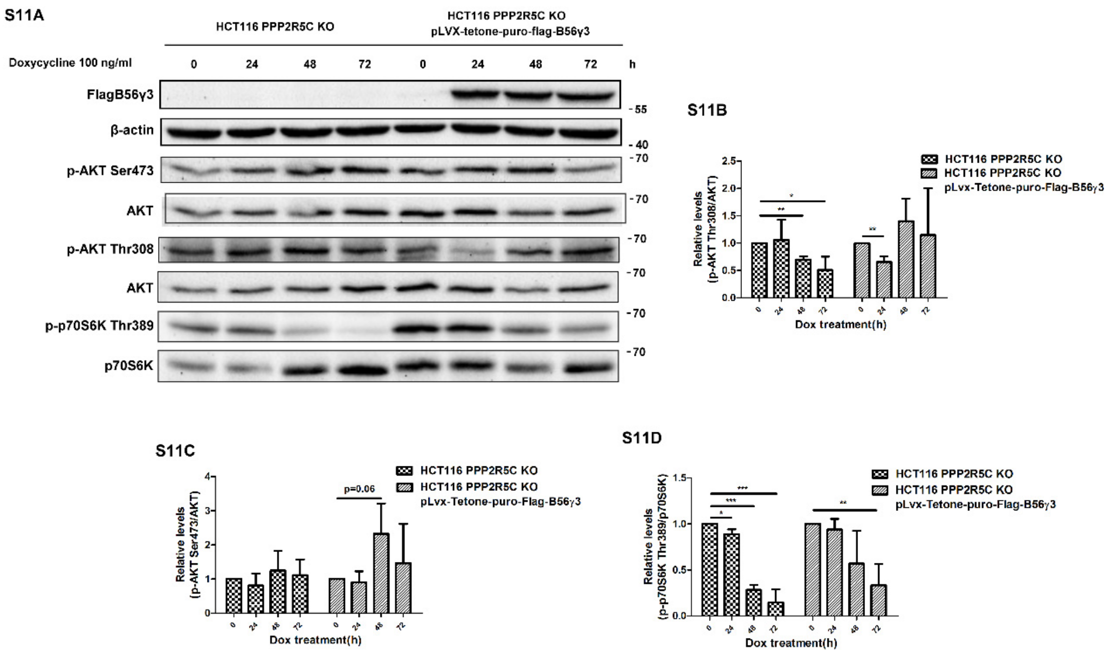

Supplementary Figure 11. The upregulation of AKT phosphorylation by re-expression of B56γ3 in a PPP2R5C knockout clone of HCT116 cells occurred at 48 h after B56γ3 re-expression. (A) Lysates of a PPP2R5C knockout clone of HCT116 cells and a PPP2R5C knockout clone of HCT116 cells harboring pLVX-tetone-puro-Flag-B56γ3 treated with doxycycline (100 ng/ml) at indicated time points were analyzed by SDS-PAGE and Western blotting using antibodies as indicated. Representative blots of at least three independent experiments with similar results were shown. **(B-D)** Graphs shown are the mean relative expression levels of phospho-Akt (Thr308 and Ser473) and phospho-p70S6K (Thr389) of three independent experiments with similar results. The differences were assessed for statistical significance by student's t test with p value (*(<0.05), **(<0.01), ***(<0.001)). (n=3)

References

1. Tang, Z., et al., GEPIA: a web server for cancer and normal gene expression profiling

and interactive analyses. Nucleic Acids Res, 2017. 45(W1): p. W98-W102.
